# Unlocking potent anti-tuberculosis natural products through structure–activity relationship analysis

**DOI:** 10.1007/s13659-025-00529-4

**Published:** 2025-07-07

**Authors:** Delfly Booby Abdjul, Fitri Budiyanto, Joko Tri Wibowo, Tutik Murniasih, Siti Irma Rahmawati, Dwi Wahyu Indriani, Masteria Yunovilsa Putra, Asep Bayu

**Affiliations:** 1 Research Center for Vaccine and Drugs, Research Organization for Health, National Research and Innovation Agency (BRIN), Jalan Raya Jakarta Bogor KM.46, Cibinong, Bogor, West Java 16911 Indonesia; 2 North Sulawesi Research and Development Agency, Jalan 17 Agustus, Manado, North Sulawesi 95116 Indonesia

**Keywords:** Tuberculosis, Natural Products, Structure–activity relationship, Anti-TB scaffold

## Abstract

Tuberculosis (TB) remains a world health problem due to the high number of affected individuals, high mortality rates, prolonged treatment durations, and the increasing prevalence of resistance to commercial TB drugs. The emergence of resistance to anti-TB drugs has necessitated urgent research into drug discovery and development, focusing on novel mechanisms of action against *Mycobacterium tuberculosis* resistant strains. Natural products, with their remarkable structural diversity and bioactivity, are promising sources for the development of new TB drugs or the identification of potential chemical scaffolds exhibiting potent and novel biological activity with minimal or no cytotoxicity to host cells. This review focuses on potent anti-TB natural products with minimum inhibitory concentration (MIC) values below 5 µg mL^–1^ and examines their structure–activity relationship (SAR). Significant characteristics and relevant biological properties of each compound were analysed using a Random Forest, machine learning algorithm, to explore SAR. Using molecular docking, AutoDock Vina was utilised to assess molecular interactions with protein targets, and predictive accuracy was enhanced using the XGBoost machine learning model. These analyses provide insights into the mode of action of these compounds and help identify key structural features contributing to their anti-TB activity. In addition, this review examines the correlation between the potency of selected anti-TB compounds and their cytotoxicity, offering valuable insights for the identification of promising scaffolds in TB drug discovery.

**Graphical Abstract:**

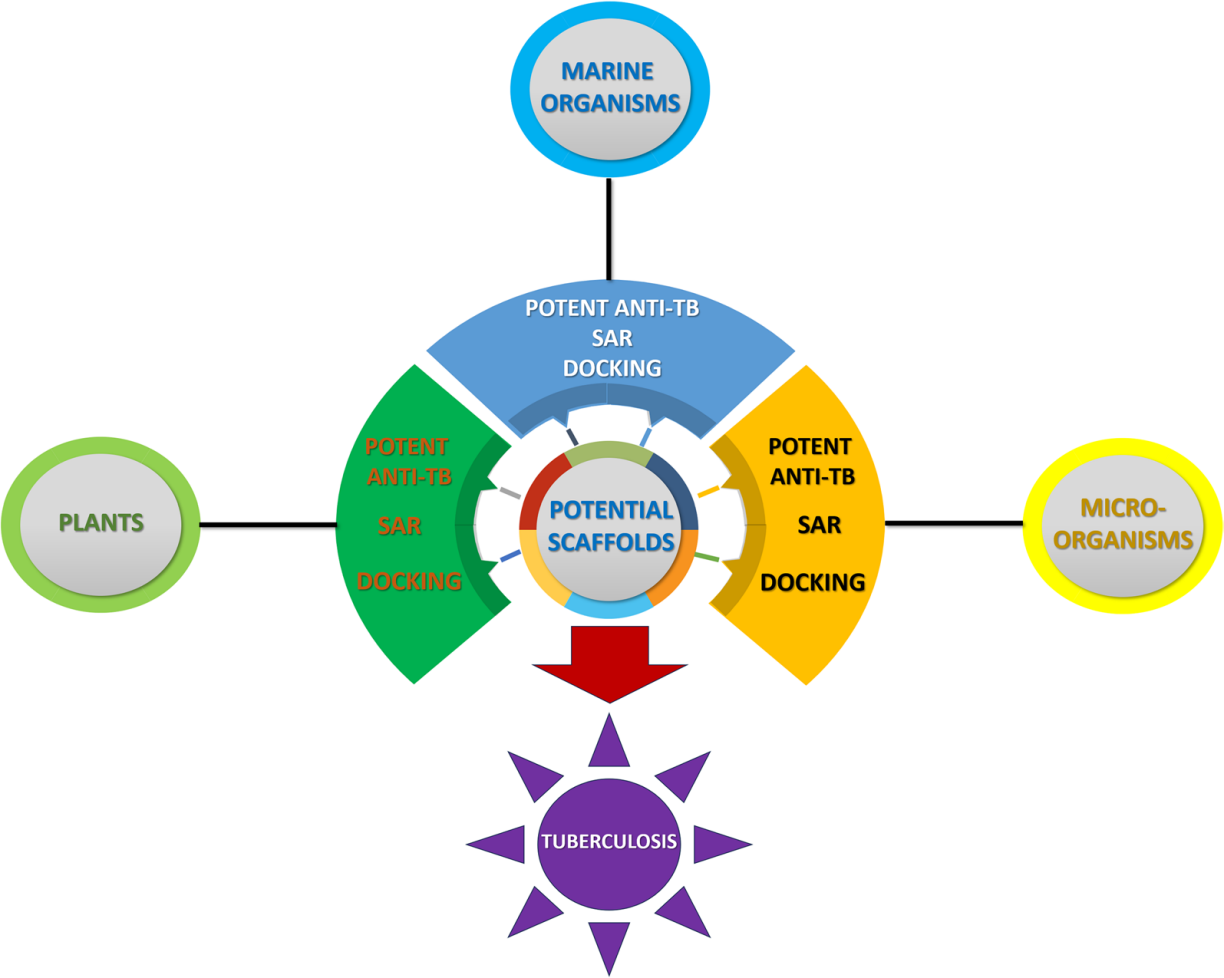

**Supplementary Information:**

The online version contains supplementary material available at 10.1007/s13659-025-00529-4.

## Introduction

Tuberculosis (TB) is an infectious disease caused by *Mycobacterium tuberculosis.* Although current standard treatments for TB are effective, the disease remains a significant world health problem due to its high prevalence, high mortality rates, and prolonged duration of treatment, particularly in developing countries [1–3]. In 2021, approximately 10.6 million people were affected by TB, resulting in 1.6 million deaths worldwide [4]. Consequently, TB is the second-leading infectious disease in terms of mortality, following COVID-19. According to the World Health Organization (WHO), the majority of TB cases occur in Asia (58%) and Africa (28%) while the remaining 14% are distributed across the rest of the world [5]. 

The situation is further complicated by the emergence of multidrug-resistant (MDR-TB) and extensively drug-resistant (XDR-TB) strains of *M. tuberculosis* [6–8]*.* For instance, about 450,000 cases of rifampicin-resistant TB were reported in 2021, representing a 3% rise in the incidence of drug-resistant TB from 2020 [4]. This growing resistance underscores the urgent need to discover and develop novel anti-TB agents, particularly those effective against drug-resistant M. tuberculosis strains.

Natural products have long been recognized as valuable sources for drug discovery due to their immense structural diversity and broad bioactivity. Between 1981 and 2004, approximately 70% of the 1562 newly approved pharmaceuticals were derived from natural sources [9]. In 2019, nine of the 38 small-molecule drugs approved by the US Food and Drug Administration (FDA) were also of natural origin [10]. Natural products have played a crucial role in the discovery of anti-TB drugs. Although many investigational anti-TB agents currently in clinical trials are synthetic, four of the first-line anti-TB drugs (ethambutol, isoniazid, pyrazinamide, and rifampicin) were developed from natural sources [11, 12]. Rifampicin was first isolated from the actinomycete *Amycolatopsis mediterranei* [13]. The MIC values of isoniazid and rifampicin against the MDR strain of *M. tuberculosis* were 2–4 and 1 µg mL^–1^, respectively [14]. While the MIC of ethambutol was 2.5 µg mL^–1^ [15], and pyrazinamide was 6 to 50 µg mL^−1^ [16]. The several second-line anti-TB drugs derived from natural resources are cycloserine (isolated from *Streptomyces orchidaceous*; MIC: 4 µg mL^–1^), streptomycine (*Streptomyces griseus*; MIC 0.5–2 µg mL^–1^), amikacin (*Streptomyces griseus;* MIC: 0.25–1 µg mL^–1^), kanamycin (*Streptomyces griseus*; MIC: 2.5 µg mL^–1^), capreomycin (*Streptomyces capreolus;* MIC: 1–2.5 µg mL^–1^), and clarithromycin [17–21]. These successes can be attributed to the unique bioactive sites of natural compounds, which often exhibit potent inhibition and novel mechanisms of action against *M. tuberculosis*.

The exploration of natural products for anti-TB drug discovery is guided by structure–activity relationship (SAR) analysis, which establishes the correlation between a compound’s chemical structure and its biological activity [22, 23]. This approach provides critical structural insights that facilitate the rational design of optimised compounds with targeted mechanisms of action [24, 25]. Given that *M. tuberculosis* exhibits resistance not only to long-established drugs but also to recently developed therapies, SAR studies are essential for identifying novel structural features that enhance drug efficacy. For instance, resistance to the macrolide class of antibiotics has been linked to their low permeability across the *M. tuberculosis* cell wall, thus hindering the drug’s efficacy. By leveraging SAR, researchers can optimize drug candidates to improve alignment with novel therapeutic targets and the likelihood of success in combating *M. tuberculosis* resistance.

**Fig. 1:**
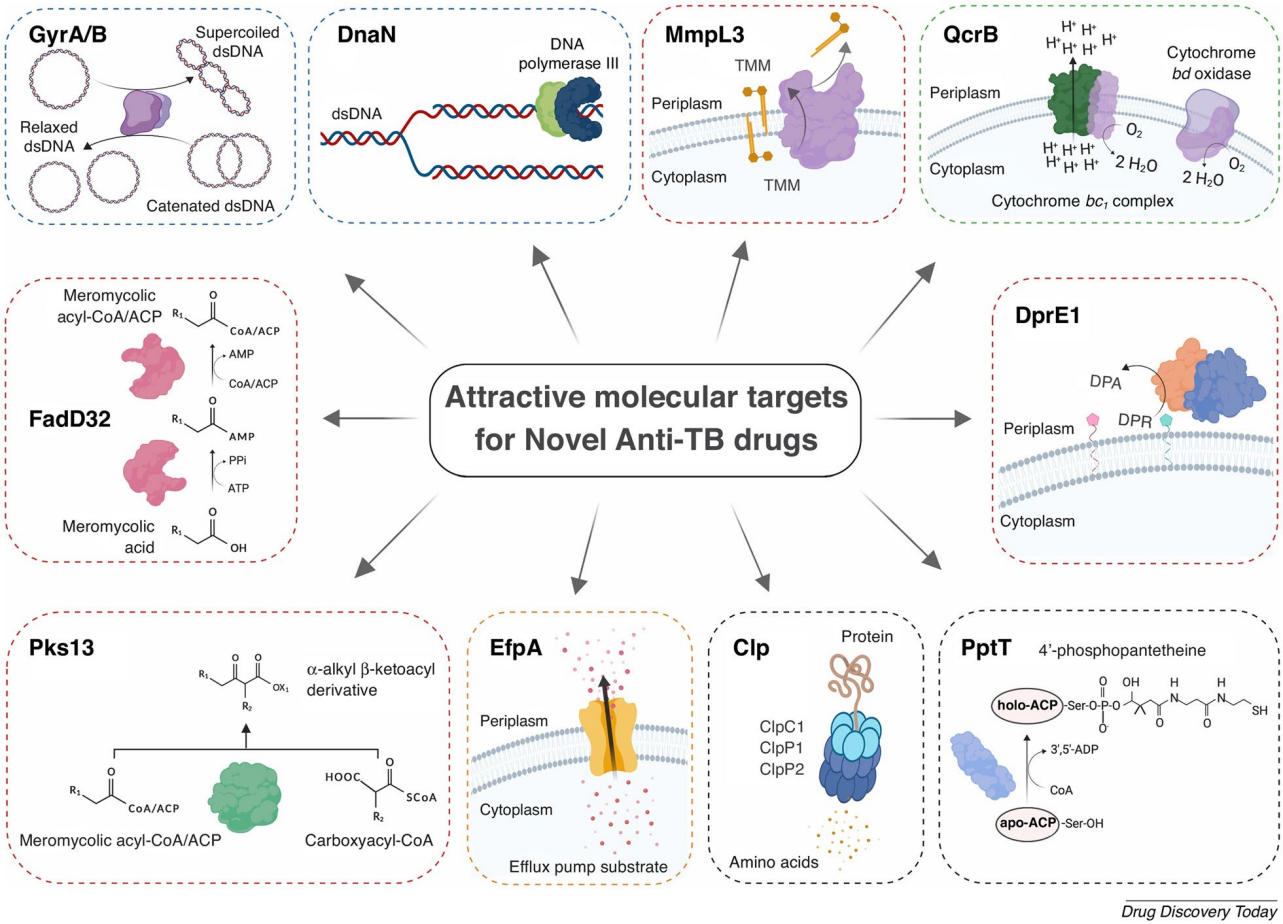
Attractive molecular targets for antitubercular drugs. Reprint with permission from Huszár et al. [1]

Currently, the design of new TB drugs focuses on targeting essential bacterial pathways, including DNA gyrase, cell wall biosynthesis, oxidative phosphorylation, efflux pumps, and intermediary metabolism, to disrupt critical functions of *M. tuberculosis* and counteract resistance mechanisms (Fig. 1) [26].

**Fig. 2:**
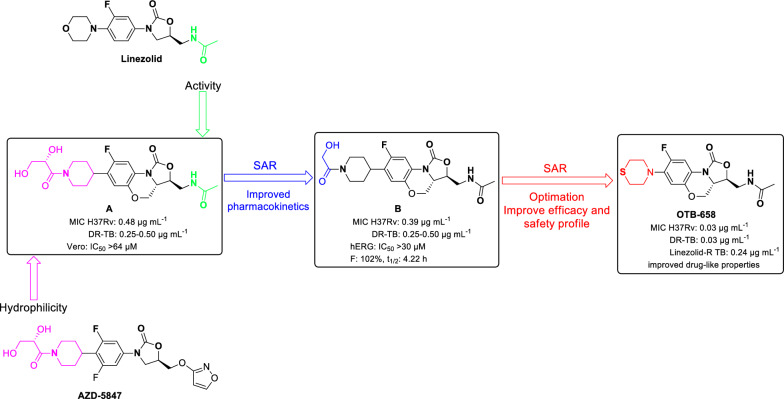
Development of OTB-658 through SAR study

For instance, the anti-TB agent BTZ043 (8-nitro-1,3-benzothiazin-4-one) was effective in killing *M. tuberculosis* and *M. smegmatis* with MICs of 0.001 and 0.003 µg mL^–1^, by the mechanism of deactivation DprE1 [27]. **OTB-658** emerged as a promising synthetic anti-TB drug candidate through a comprehensive SAR study (Fig. 2) and has now progressed to preclinical development (GLP Toxicology studies) [28, 29]. The development of **OTB-658** began with the discovery of fluoro-benzoxazinyl-oxazolidinone (compound **A**), which incorporates an acetylaminomethyl unit from linezolid and a dihydroxypropinoyl tetrahydropyridine moiety from **AZD-5874** [30]. The tricyclic-fused benzoxazinyl-oxazolidinone scaffold was selected as the basis for further research due to its favorable pharmacokinetic profile. Compound A demonstrated good anti-TB activity against *M. tuberculosis* H37Rv, with an MIC_90_ of 0.48 µg mL^–1^, while exhibiting low cytotoxicity to Vero cells (IC_50_ > 64 µM). Further optimization of fluoro-benzoxazinyl-oxazolidinone analogs led to the identification of compound **B**, which exhibited enhanced activity against both *M. tuberculosis* (H37R_V_) and drug-resistant strains, with MIC_90_ values of 0.39 µg mL^–1^ and 0.20–0.50 µg mL^–1^, respectively [30]. Notably, compound **B** features a hydroxyacetyl moiety attached to tetrahydropyrimidine, replacing the dihydroxypropanoyl group in **A**. This modification resulted in a favourable pharmacokinetic (PK) profile while maintaining a high safety margin, as it exhibited no significant cytotoxicity in HepG2 cells (IC_50_ > 64 µg mL^–1^) and showed minimal inhibition hERG K^+^ channel (IC_50_ > 30 µM). Subsequent optimization efforts aimed at improving efficacy and drug-like properties of fluoro-benzoxazinyl-oxazolidinone derivatives ultimately led to the discovery of **OTB-658** [28]. **OTB-658** displayed improved in vitro and in vivo drug-like properties compared with its parent linezolid, with potent anti-TB activity against both drug-susceptible *M. tuberculosis* H37Rv and drug-resistant strains, achieving an MIC_90_ of just 0.03 µg mL^–1^ [28].

**Fig. 3:**
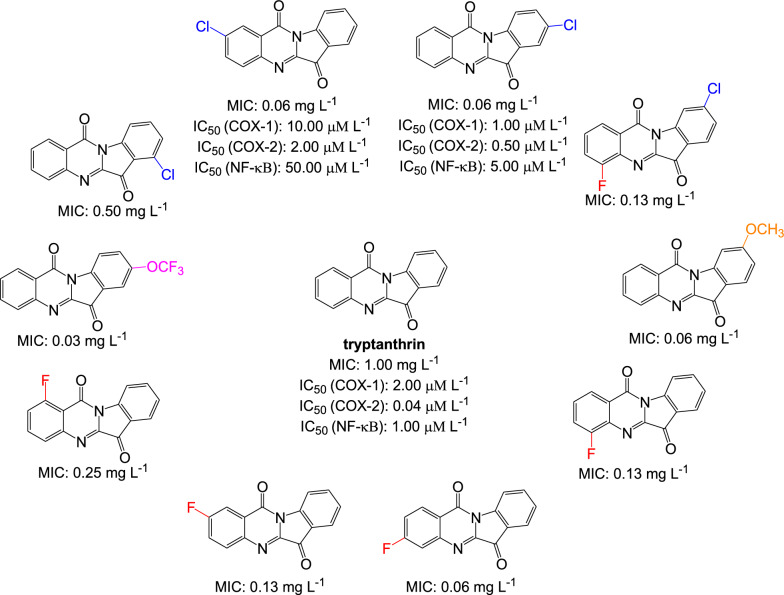
Inhibitory activities of tryptanrthrin and its derivatives on *M. tuberculosis* H37Rv and several enzymes [31]

Tryptanthrin is an indoloquinazoline alkaloid isolated from natural sources; *e.g.*, the fungus of *Candida lipolytica, Leucopaxillus cerealis*, *Schizophyllum commune* and the plants of *Couroupita guaianensis Abul*, *Strobilanthes cusia*, *Isatis indigotica*; that showed antimycobacterial activity against *M. tuberculosis* H37Rv [31]. Optimization of some functional groups attached on its parent structure enhanced the antimycobacterial activity (Fig. 3). For instances, fluorinated in the benzene ring of tryptanthrin increased the MIC value against *M. tuberculosis* from 1.00 to 0.06 mg L^−1^. An increasing MIC value was also observed when it was chlorinated. However, these chlorinated tryptanthrin exhibited slightly lower activity compared with their fluorinated derivatives. The most optimum structure was gained when substituted R8 with OCF3 with MIC value 0.03 mg L^−1^ [31].

Numerous compounds isolated from natural sources have been shown to have anti-TB properties [32–34]; however, their limited yield, cytotoxicity, and poor solubility hinder their development as clinical candidates [35–37]. Compounds with an MIC < 10 µg mL^–1^ are classified as strong anti-TB agents, while those with MIC up to 50 µg mL^–1^ are considered moderate [5].

**Fig. 4:**
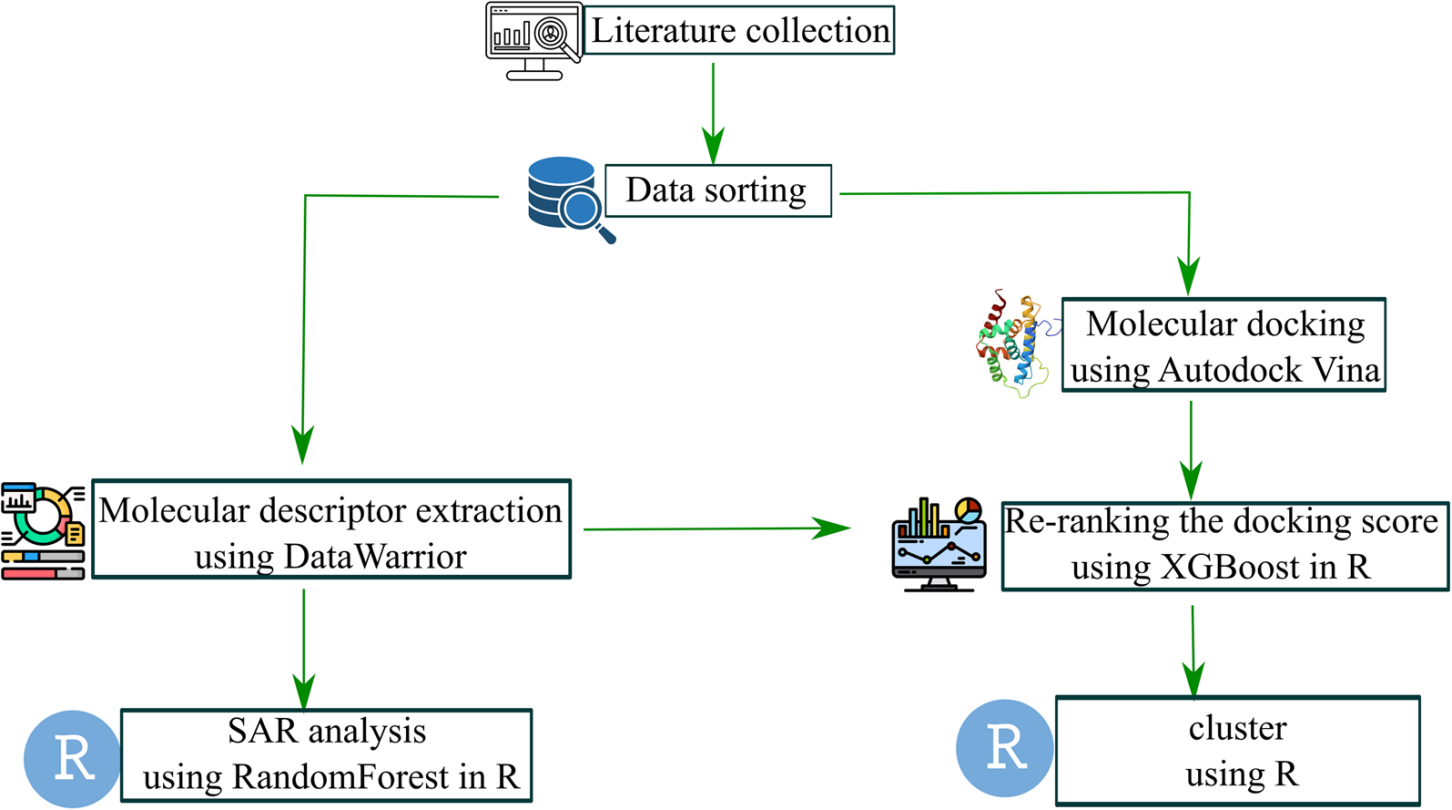
Overview of the methodology applied in this critical review. Icons sourced from Flaticon under the Basic License (CC3.0, Creative Commons)

This review focuses on the SAR of anti-TB natural products derived from marine organisms, terrestrial plants, and microorganisms, particularly those exhibiting potent efficacy against *M. tuberculosis* with MIC values < 5 µg mL^–1^. The study is categorized into two main sections: (1) data collection and refinement, and (2) predictive modelling and interaction studies (Fig. 4). Data collection was conducted using two prominent scientific databases, Scopus and PubMed, focusing exclusively on natural products exhibiting potent anti-TB activity, defined by MIC values of below 5 µg mL^–1^. Each compound’s key structural characteristics and relevant biological properties were thoroughly analyzed in this review. Following data processing, machine learning analyses were employed to explore SAR using the Random Forest algorithm. The molecular interactions of the compounds with protein targets were assessed through docking studies conducted using Autodock Vina (Table S1). To enhance the predictive accuracy of the docking results, docking scores obtained from Vina were re-ranked using the machine learning model XGBoost (Table S2). Detailed descriptions of the methodologies employed are provided in the Supplementary Materials.

These analyses offer valuable insights into the mode of action of the studied compounds and help identify key structural features essential for anti-TB activity. SAR studies can aid in rational design and synthesis of novel derivatives with improved potency and selectivity, potentially leading to more effective anti-TB agents. In addition, this review highlights the correlation between the potency and cytotoxicity of several anti-TB compounds, providing crucial guidance for selecting potential scaffolds for TB drug development.

## Potent anti-TB compounds from marine organisms

Marine organisms produce a wide variety of metabolites with diverse biological activities and unique structural features [38, 39]. Many marine natural products belong to novel chemical groups that are not found in terrestrial organisms [40, 41]. Due to their adaptation to extreme environments, such as broad thermal ranges, high pressure, and high salt concentrations, these metabolites play a crucial role in protecting marine organisms from predation and competition [42–44]. Since the ocean covers more than seventy percent of the earth’s surface, marine environments represent a highly attractive source for discovering novel natural products that could serve as candidates and leads for new drug development [45, 46].

Research into the pharmacological properties of marine-derived natural products has led to the discovery of many potent bioactive compounds with potential clinical applications [47, 48]. In recent years, many novel natural products with significant biological activities have been identified from marine organisms. According to Carroll and co-workers [49–53], 7366 new natural products with diverse biological activities were isolated from marine organisms between 2017 and 2021. Moreover, there are currently 14 FDA-approved drugs derived from marine organisms [54], with another 20 in clinical trials [55].

Given this vast potential, marine natural products also offer a promising avenue for anti-TB drug discovery. Over the past 30 years, no fewer than 176 anti-TB compounds with diverse chemical structures have been reported from marine organisms [56].

### Alkaloid

**Fig. 5:**
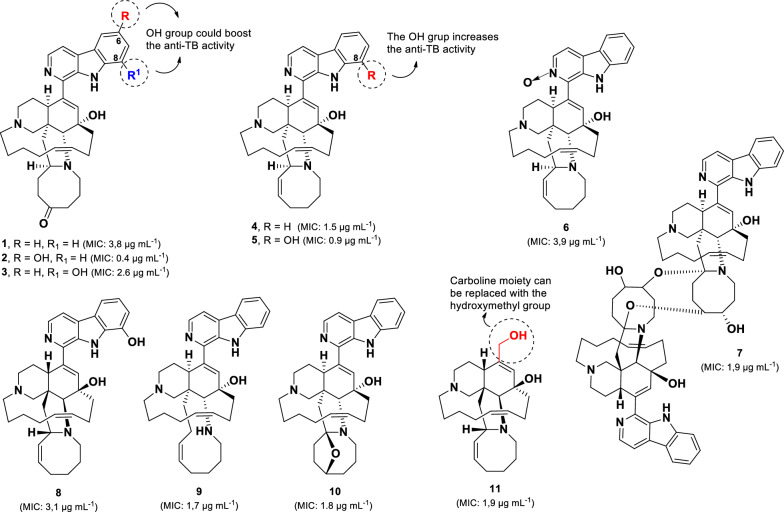
Chemical structures of manzamine alkaloids (**1**–**11**)

A group of manzamine derivatives, identified from the Indonesian marine sponge *Acanthostrongylophora* sp., demonstrated potent anti-TB activity, with ten of its compounds (**1–10**) (Fig. 5) exhibiting MIC values below 5 µg mL^–1^ [57–59]. Manzamines are unique polycyclic alkaloids isolated from marine sponges, typically characterised by a pentacyclic system with a β-carboline moiety [60, 61]. Among them, 6-hydroxymanzamine E (**2**) and 8-hydroxymanzamine A (**5**) displayed the strongest activity against *M. tuberculosis* (H37Rv), with MIC values of 0.4 µg mL^–1^ and 0.9 µg mL^–1^, respectively.

In contrast, their parent compounds, manzamine E (**1**, MIC 3.8 µg mL^–1^) and A (**4**, MIC 1.5 µg mL^–1^), exhibited lower anti-TB activity, suggesting that hydroxylation at the C-6 or C-8 position of the β-carboline core enhances potency. This observation is further supported by a comparison between **1** and its 8-hydroxy derivative (**3)**, which exhibited greater anti-TB activity (MIC 2.6 µg mL⁻^1^) than **1**, indicating that hydroxylation at C-8 also contributes to activity. Moreover, compound **6** (MIC 3.9 µg mL^–1^) demonstrated that introducing an *N*-oxide in the β-carboline moiety decreased the anti-TB potency compared to its parent compound **4**. Similarly, compound **8** (MIC 3.1 µg mL^–1^) exhibited three times less activity than its enantiomer **5**, suggesting that the stereochemistry of **5** is more favourable for anti-TB activity.

Interestingly, the dimeric form of manzamine (**7**, MIC 1.9 µg mL^–1^) displayed comparable anti-TB activity to its monomeric counterparts (**4**, **9,** and **10**), indicating that dimerization does not significantly enhance potency. Additionally, variations in the eight-membered ring, as observed in **4**, **9**, and **10**, did not substantially affect anti-TB activity. However, the presence of a ketone group in this ring, as in compound **1**, reduced activity by approximately twofold.

Notably, ircinol A (**11**, MIC 1.9 µg mL^–1^) exhibited similar anti-TB activity to compound **4** (Fig. 5) [58], suggesting that the β-carboline moiety can be effectively replaced by a hydroxymethyl group without significantly compromising potency.

**Fig. 6:**
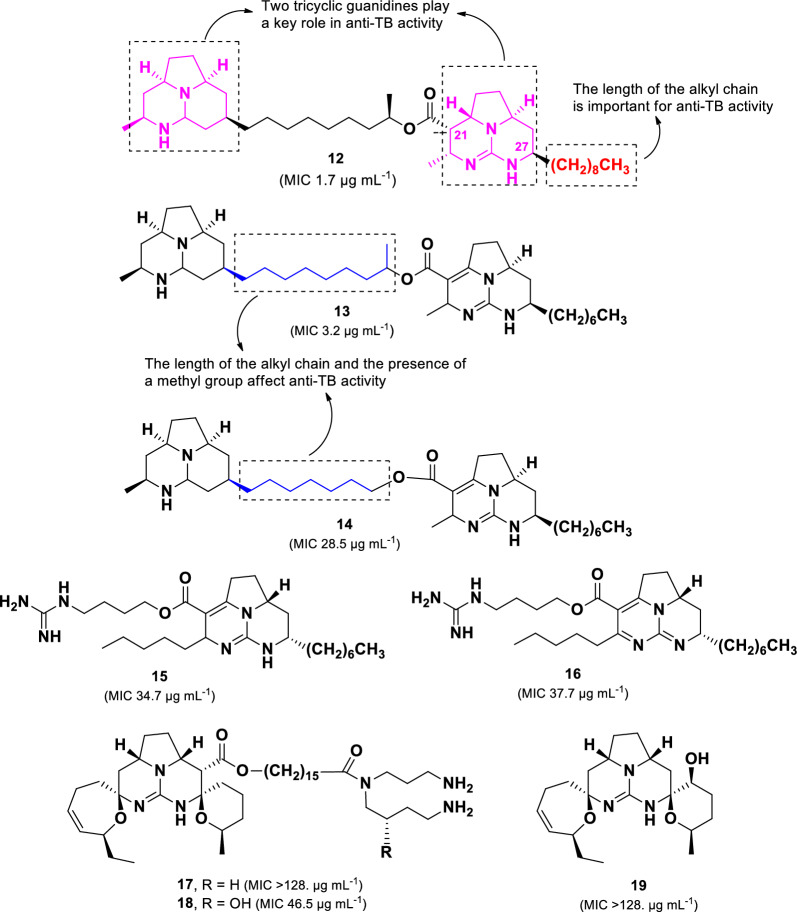
Chemical structures of guanidine alkaloids (**12**–**19**)

Eight polycyclic guanidine alkaloids (**12**–**19**) (Fig. 6) isolated from the Jamaican marine sponge *Monanchora unguifera* were evaluated for their anti-TB activity [62]. Among them, batzelladines L (**12**, MIC 1.68 µg mL^–1^) and N (**13**, MIC 3.18 µg mL^–1^) exhibited strong activity against *M. tuberculosis* H37Rv*.* Batzelladines belong to a class of tricyclic guanidine alkaloids, typically linked to another guanidine moiety via an ester bond [63]. The anti-TB activity of **12** and **13** was greater than **15**–**19**, indicating that the presence of two tricyclic guanidine units enhances anti-TB activity compared to structures containing only one tricyclic guanidine group. In addition, the short alkyl chain and absence of a methyl group on the aliphatic bridge, as observed in compound **14** (MIC 28.5 μg mL^–1^), resulted in a nine-fold decrease in anti-TB activity. Although compounds **12** and **13** share structural similarities, compound **12** exhibited twice the potency of **13**, suggesting that the bonding type at C-21/22 and the length of the alkyl chain at C-27 play crucial roles in anti-TB activity.

**Fig. 7:**
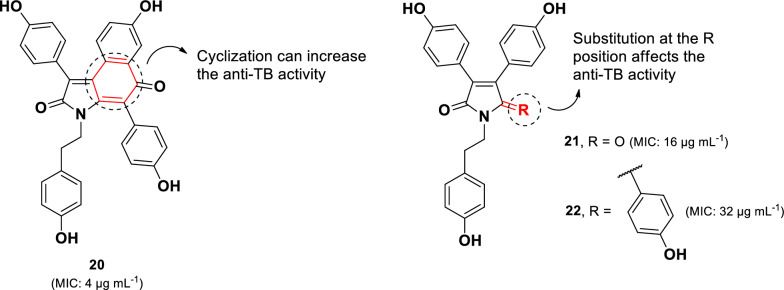
Chemical structures of pyrrole alkaloids (**20**–**22**)

Another class of anti-TB alkaloids containing the 3,4-diaryl pyrrole core (**20–22**) (Fig. 7) has been isolated from the Indian marine sponge *Dendrilla nigra* [64]. Among these, denigrin C (**20**) exhibited the most potent activity against *M. tuberculosis* H37Rv, with an MIC value of 4 µg mL^–1^, compared to its analogs denigrins A (**21**, MIC 16 µg mL^–1^) and B (**22**, MIC 32 µg mL^–1^). The superior activity of denigrin C (**20**) relative to denigrin B (**22)**, a closely related structural analog, suggests that modification of the pyrrole and hydroxyphenyl rings to form the cyclohexenone ring enhances anti-TB activity. Furthermore, a structural comparison of denigrins A (**21**) and B (**22**) indicates that replacing a carbonyl group in the pyrrole unit with a hydroxyphenyl group leads to an approximately twofold decrease in activity. Recently, four new denigrin derivatives (D-G), along with the previously known denigirins A and B, were identified from the Maldives marine sponge *Dactylia* sp [65]. However, these denigrin derivatives did not show activity against the PAX3-FOXO1 fusion gene associated with Rhabdosarcoma.

**Fig. 8:**
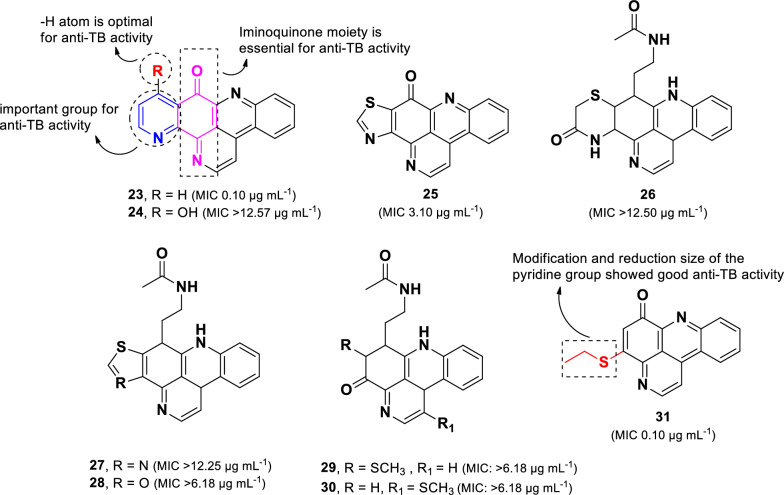
Chemical structures of pyridoacridine alkaloids (**23**–**31**)

Ascididemin (**23**), a unique alkaloid belonging to the pyridoacridine class [66], was first isolated as an anti-leukemic agent from the Okinawan tunicate *Didemnum* sp [67]. Among evaluated compounds, ascididemin (**23)**, exhibited the highest activity against *M. tuberculosis* H37Rv (MIC 0.10 µg mL^–1^), followed by kuanoniamine A (**25**, MIC 3.10 µg mL^–1^). In contrast, other related compounds (**26–30)** displayed weak activity, with MIC values exceeding 6.18 µg mL^–1^ (Fig. 8). Structural comparisons among these compounds (**23**–**30**) revealed that the intact iminoquinone moiety, as observed in **23** and **24**, is essential for the anti-TB activity. Despite its structural similarity to **23**, compound **24** (MIC > 12.57 µg mL^–1^) exhibited a significant loss of activity, likely due to hydroxylation at C-11. Additionally, the pyridine moiety in **23** was found to be more favorable for anti-TB activity than the thiazole in **25**. Further synthetic modifications of **23** led to the discovery of compound **31** (MIC 0.10 µg mL^–1^) (Fig. 8), which demonstrated comparable anti-TB activity to ascididemin (**23**) but with reduced cytotoxicity. These findings suggests that modification to the pyridine ring and a reduction in the overall molecular size of ascididemin (**23**) could serve as a promising strategy for future anti-TB drug development [66].

### Terpene

**Fig. 9:**
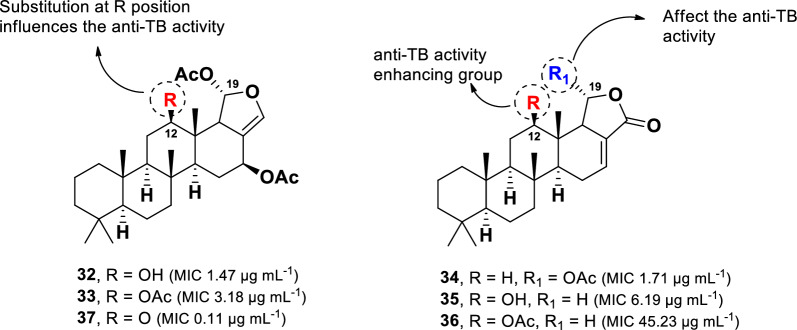
Chemical structures of scalarane sesterterpenes (**32**–**37**)

A chemical investigation of the Thai marine sponge *Brachiaster* sp. led to the isolation of anti-TB scalarane-type sesterterpenes (Fig. 9) [68]. These compounds feature a fused ring system of 6/6/6/6-tetracyclic or 6/6/6/6/5-pentacyclic [69]. Among them, heteronemin (**32**) and 12-deacetoxyscalarin-19-acetate (**34**) exhibited the most potent activity, with MIC values of 1.47 µg mL^–1^ and 1.71 µg mL^–1^, respectively. Other derivatives, including heteronemin acetate (**33**), 12-deacetyl-12-epi-19-deoxyscalarin (**35**), and 12-epi-19-deoxyscalarin (**36**), inhibited *M. tuberculosis* growth with MIC values of 3.18 µg mL^–1^, 6.19 µg mL^–1^, and 45.23 µg mL^–1^, respectively.

Heteronemin (**32**), originally discovered from the marine sponge *Heteronema erecta*, was the first scalarane-type sesterterpene reported to exhibit anti-TB activity. A structural comparison of compounds **32–36** highlights two key structural features influencing anti-TB activity: the presence of an acetoxy group at C-19 and the nature of the substituent at C-12. Compounds **32–34**, which contain an acetoxy group linked to C-19, exhibited significantly stronger anti-TB activity than related compounds lacking this moiety (**35** and **36**). This trend is further supported by the comparison of **34** and **35**, where the presence of an acetoxy group at C-19 in **34** enhances activity fourfold compared to **35**, a which lacks this functional group. Furthermore, the replacement of a hydroxyl in **32** with an acetoxy moiety, as observed in **33**, resulted in a two-fold decrease in activity. A similar pattern was observed in the comparison of **35** and **36**, underscoring the importance of the substituent at C-12 for anti-TB activity. Further studies on a series of semi-synthetic derivatives identified a heteronemin (**32**) derivative featuring a ketone unit at C-12 (**37**) (Fig. 9), which exhibited enhanced anti-TB activity with an MIC value 0.11 µg mL^–1^, surpassing the potency of **32** [70].

## Potent anti-TB from terrestrial plants

Plants have been used as natural sources of medicine since ancient times [71, 72]. Their utilization has evolved over generations, influenced by local wisdom [73, 74]. Advances in medicine and chemistry have facilitated the discovery of new plant-derived pharmaceuticals [75–77], such as silymarin for heart disease, paclitaxel for cancer, and artemisinin for malaria [78, 79]. In the early twenty-first century, 11% of WHO-categorized essential and basic medicines were derived from flowering plants [80]. Over the past few decades, various plants have been examined to identify novel bioactive compounds. To date, approximately 200,000 plant-derived compounds have been documented, some of which have displayed anti-TB activity [81].

Several structures derived from plants have been used for anti-TB drugs were phloretin (derived from apple peel; MIC_90_ M.tb. 182 µM), tryptanthrin (*Wrightia hanleyi*; MIC 1 µg mL^−1^), β-elemene (*Curcuma longa*; MIC 32 µg mL^−1^), R-limonen (orange peel; MIC 32 µg mL^−1^), bisabolol (*Matricaria chamomilla*; MIC 64 µg mL^−1^), sabinene (Marjoram; MIC 64 µg mL^−1^), pisonin B (*Pisonia aculeate*; MIC 25 µg mL^−1^), pisonivanone (MIC 12.5 µg mL^−1^), albanols A-B (*Uvari alba*; MIC 26 µM), artesunate (*Artemesia annua;* MIC 75 µM, artemisin (*Artemesia annua*; MIC 75 µM) [17].

### Alkaloid

**Fig. 10:**
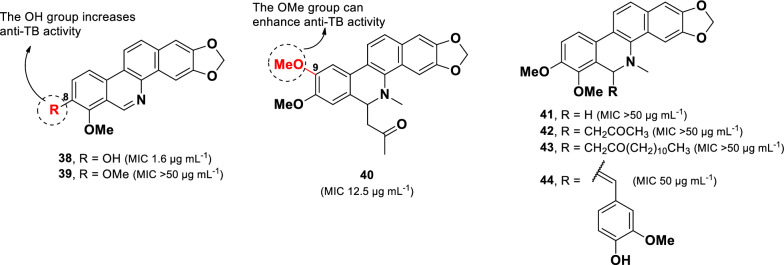
Chemical structures of benzophenanthridine alkaloids (**38**–**44**)

A chemical study of medicinal plants used in Mozambique to treat TB and respiratory disorders led to the identification of several plant species with notable anti-TB activity [82]. Among them, *Zanthoxylum capense* emerged as the most promising, with MIC values of 31.2–125 µg mL^–1^ against various mycobacterial strains [82]. Further phytochemical analysis of *Z. capense* roots resulted in the isolation of benzophenanthridine alkaloids (**38**–**44**) (Fig. 10) [83, 84]. Benzophenanthridine alkaloids belong to the isoquinoline class and are commonly found in plants from the Papaveraceae, Corydalis, and Rutaceae families [85].

Among the isolated compounds, decarine (**38**) displayed the highest anti-TB potency against *M. tuberculosis* H37Rv, with an MIC value of 1.6 μg mL^–1^. In contrast, norchelerythrine (**39)** exhibited weak activity (MIC > 50 µg mL^–1^) despite its structural similarity to **38**. This disparity was attributed to the replacement of the hydroxyl group with a methoxy group at C-8 in **39**. However, compounds **39** and **41** showed comparable MIC values, suggesting that the presence of *N*-methyl in **41** did not significantly influence anti-TB activity. Moreover, 6-acetonyldihydronitidine (**40**, MIC 12.5 µg mL^–1^), which contains two methoxy units at C-8 and C-9, showed stronger anti-TB activity than compound **42** (MIC > 50 µg mL^–1^), which features methoxy groups at C-7 and C-8. This finding indicates that the position of the methoxy groups plays a critical role in modulating activity. Compounds **41**–**44** share a similar core structure but differ in the functional group attached to C-6. However, these structural modifications did not enhance their anti-TB activity.

**Fig. 11:**
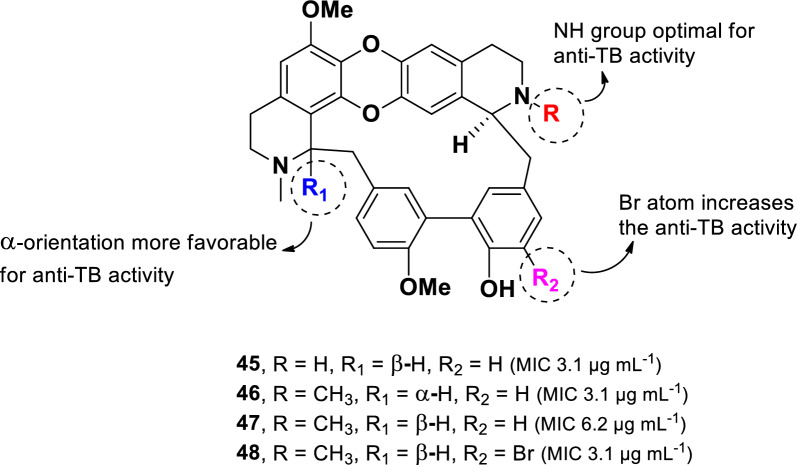
Chemical structures of bisbenzylisoquinoline alkaloids (**45**–**48**)

Other alkaloid compounds with potent anti-TB activity were isolated from a Thai edible plant *Tiliacora triandra* [86]. Three naturally occurring bisbenzylisoquinoline alkaloids (**45**–**47**) together with a synthetic derivative (**48**) (Fig. 11) were evaluated against drug-susceptible *M. tuberculosis* as well as numerous multiple multidrug-resistant (MDR-TB) isolates. Bisbenzoquinoline alkaloids are characterized by two benzylisoquinoline moieties connected through benzyl phenyl ether, diphenyl ether, or biphenyl linkages [87].

Among the tested compounds, 2′-nortiliacorinine (**45**), tiliacorine (**46**), and 13′-bromo-tiliacorinine (**48**) exhibited significant inhibitory activity against *M. tuberculosis* H37Rv, each with an MIC of 3.1 µg mL^–1^. In contrast, tiliacorinine (**47**) exhibited lower potency, with an MIC of 6.2 µg mL^–1^. The absence of an N-methyl group at N-2′ in **45** resulted in higher activity compared to **47**, suggesting that the additional ***N***-methyl group in **47** is responsible for the twofold decrease in potency. Conversely, the incorporation of a bromine atom in **48** enhanced potencies by twofold compared to its parent compound **47**. Additionally, stereochemical configuration at H-1 was found to influence anti-TB activity within this class of alkaloids. The α-orientation observed in **46** was more favorable, leading to a twofold increase potency compared to the β-orientation present in **47**. Moreover, compounds **45**–**48** showed promising activity against particular MDR-MTB isolates, with MICs values ranging from 0.7 to 6.2 µg mL^–1^.

**Fig. 12:**
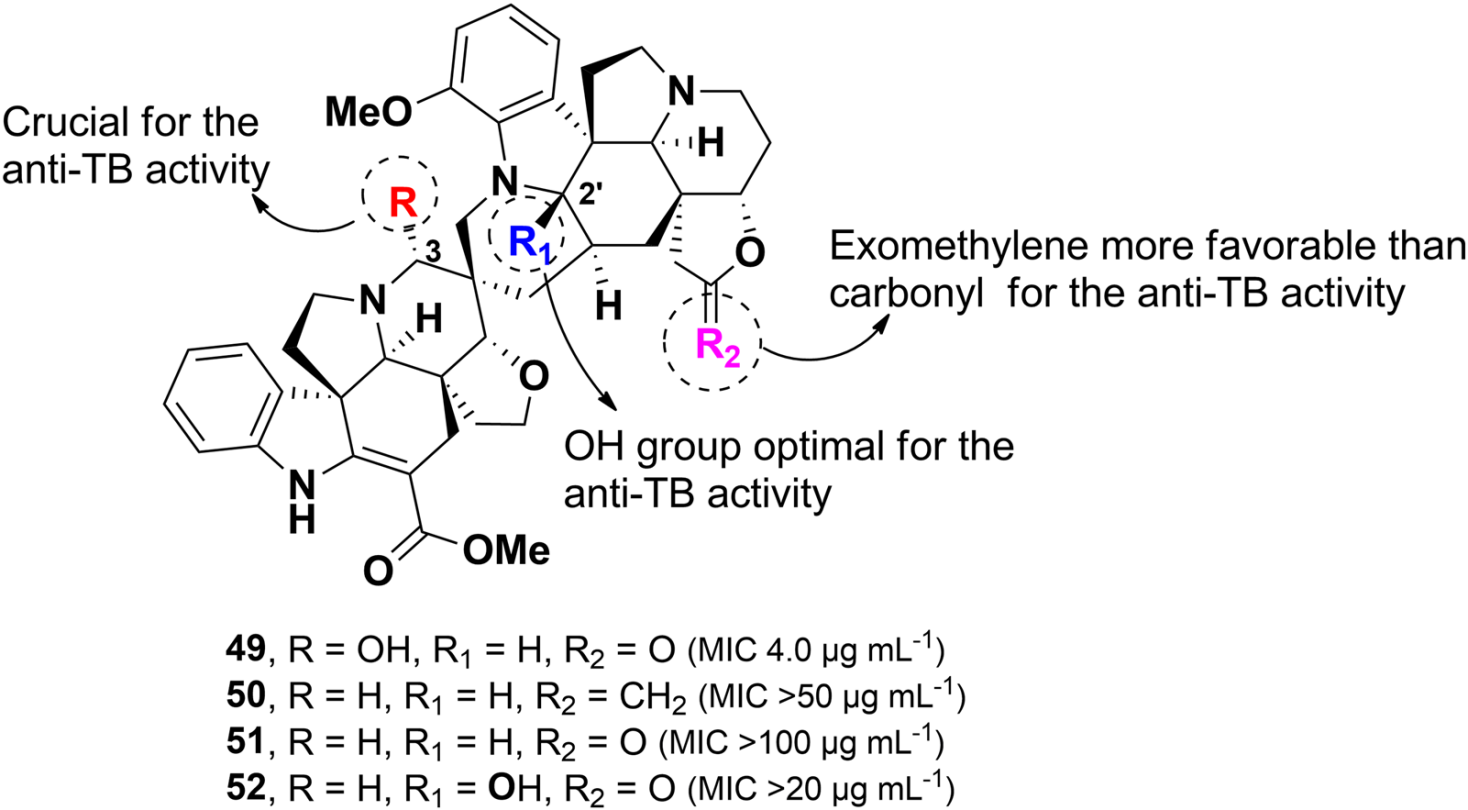
Chemical structures of spirobisindole alkaloids (**49**–**52**)

Purification of the Philippine plant *Voacanga globosa* using bioassay-guided fractionation for anti-TB activity led to the isolation of four spirobisindole alkaloids: globospiramine (**49**), deoxyvobtusine (**50**), deoxyvobtusine lactone (**51**), and vobtusine lactone (**52**), along with a triterpenoid compound (Fig. 12) [88]. Among these, compound **49** exhibited potent inhibitory activity against *M. tuberculosis* (H37Rv), with an MIC value of 4.0 µg mL^–1^. However, despite their structural similarities, the closely related compounds **50–52** showed no observable anti-TB activity. This difference in bioactivity is attributed to key structural modifications, including: (i) the substitution of a carbonyl group with an exomethylene moiety in **50,** (ii) the absence of a hydroxyl group at C-3 in **51**, and (iii) hydroxylation at C-2′ in **52**. A comparative analysis of structures **49–52** suggests that the hydroxyl group at C-3 plays a crucial role in the anti-TB activity of this spiroindole alkaloid class. Additionally, globospiramine **49** demonstrated modest cytotoxic activity against lymphocytic (HL-60) and promyelocytic (Jurkat) cell lines, with CC_50_ values of 0.75 and 0.50 µg mL^–1^, respectively. In contrast, compounds **50** and **52** were found to be non-cytotoxic [89].

### Terpene

**Fig. 13:**
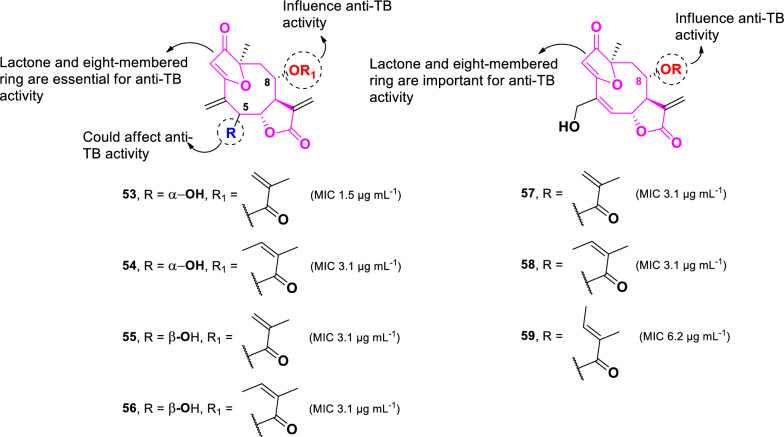
Chemical structures of sesquiterpene lactones (**53**–**59**)

A group of sesquiterpene lactones with potent anti-TB activity was isolated from *Camchaya calcarea,* a plant collected from Ubon Ratchatcani, Thailand (Fig. 13) [90]. Among these, isogoyazensolide (**53**, MIC 1.5 µg mL^–1^) showed the highest activity against *M. tuberculosis* H37Ra. Other compounds including, isocentratherin (**54**), 5-epi-isogoyazensolide (**55**), 5-epi-isocentratherin (**56**), goyazensolide (**57**), tratherin (**58**), and lychnophorolide B (**59**), demonstrated weaker activity, with MIC values of 3.1, 3.1, 3.1, 3.1, 3.1, and 6.2 µg mL^–1^, respectively. Compound **54**, which differs only in the functional group attached to C-8 of **53**, shows twofold weaker activity than **53**. Therefore, the methacryloxy group, observed in **53**, is more favorable for anti-TB activity than the angeloyloxy group in **54**. While the stereochemistry of the hydroxyl group at C-5 does not significantly influence the anti-TB activity of the angeloyloxy derivatives (**54** and **56**), it plays a crucial role in methacryloxy analogs. The α-orientation of the hydroxyl group in **53** increases activity by twofold compared to its epimer **55**. Furthermore, compounds **57–59** demonstrated comparable anti-TB activity to **53–56** despite having slight structural differences due to the substitution of exomethylene at C-4 with hydroxymethyl and the absence of a hydroxyl unit at position C-5. These findings suggest that the core sesquiterpene skeleton, particularly the lactone and eight-membered-ring heterocycle moiety, is essential for anti-TB activity.

### Naphthoquinone

**Fig. 14:**
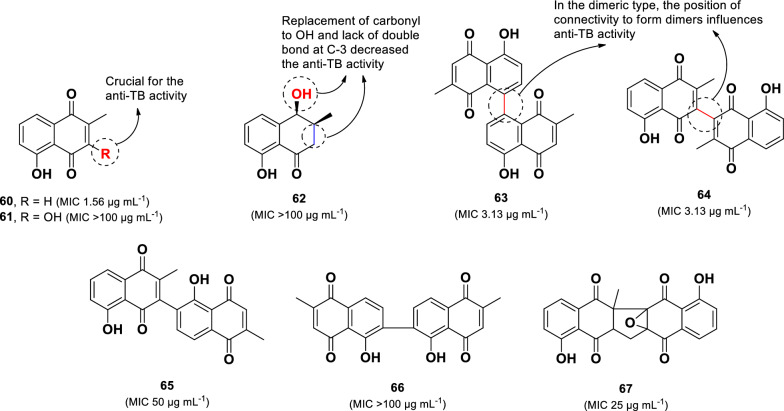
Chemical structures of naphthoquinones (**60**–**67**)

The stem bark of the Mexican endemic plant *Diospyros anisandra* yielded three monomeric (**60**–**62**) and five dimeric (**63**–**67**) naphthoquinone compounds (Fig. 14) [91]. Among them, the monomeric compound plumbagin (**60**) displayed the strongest anti-TB activity against *M. tuberculosis* H37Rv, with an MIC value of 1.56 µg mL^–1^. Two dimeric compounds, maritinone (**63**, MIC 3.13 µg mL^–1^) and 3,3′-biplumbagin (**64**, MIC 3.13 µg mL^–1^), also demonstrated significant activity. In contrast, the structurally related monomeric compounds droserone (**61**, MIC > 100 µg mL^–1^) and *cis*-isoshinanolone (**62**, MIC > 100 µg mL^–1^) were inactive in this experiment. This suggests that hydroxylation at C-3 in **61** and the modification of the carbonyl group to a hydroxyl, along with the absence of a double bond at C-3 in **62,** are responsible for their lack of anti-TB activity. Among the dimeric compounds, compounds **63** and **64** exhibited potent anti-TB activity, whereas chitranone (**65**, MIC 50 µg mL^–1^) and elliptinone (**66**, MIC > 100 µg mL^–1^) were significantly less active. This suggests that the connectivity pattern forming the dimeric structures plays a crucial role in determining their activity. Additionally, zeylanone epoxide (**67**) showed only moderate activity (MIC 25 µg mL^–1^), suggesting that the presence of epoxide and cyclopentane groups in its structure does not increase anti-TB activity. A similar activity pattern was observed when these compounds were tested against the MDR-TB (strain CIBIN 99), further confirming the structure–activity relationship findings.

## Potent anti-TB from microorganisms

Microorganisms are a valuable resource for discovering novel drugs to combat emerging diseases due to their rich diversity in bioactive compounds [92, 93]. The search for natural products derived from microorganisms origin has expanded significantly since the 1970s, driven by advancements in purification techniques, spectroscopy analysis, and integrative approaches that combine chemical evaluation with genetic information [94–96]. The successful discovery of several revolutionary drugs such as penicillin, streptomycin, and rifampicin [26, 97], highlights the immense potential of microbial sources in drug development. Recently, there has been a growing interest natural products derived from microorganisms, particularly those associated with plants and invertebrates from both marine and terrestrial habitats [98–100]. This exploration has led to the identification of promising anti-TB compounds [101], further reinforcing the importance of microorganisms in the search for novel therapeutic agents.

### Alkaloid

**Fig. 15:**
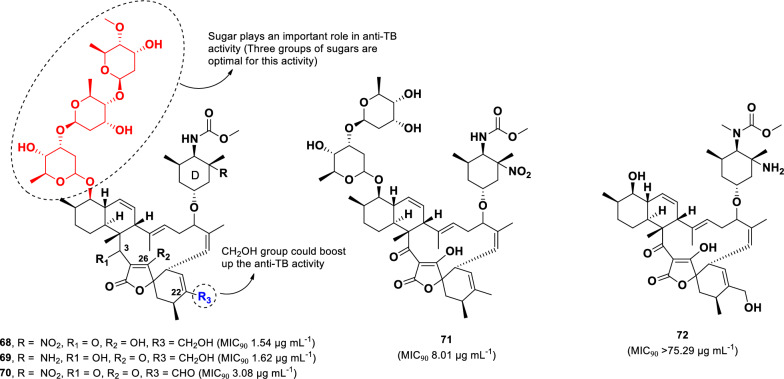
Chemical structures of spirotetronates (**68**–**72**)

Five spirotetronate compounds (**68**–**72**) were isolated from *Streptomyces* sp. (1053U.I.1a.3b) (Fig. 15), which was cultured from the Philippine conoidean mollusk *Lienardia totopoten* [102]. Spirotetronates are characterized by a structural linkage between a cyclohexane or cyclohexene and a tetronic acid spiro moiety [103]. Among the isolated compounds, lobophorins B (**68**) and C (**69**) exhibited the highest anti-TB activity against *M. tuberculosis* (H37Ra), with MIC_90_ values of 1.54 µg mL^–1^ and 1.62 µg mL^–1^, respectively. These two compounds differ structurally in the C-3, C-26, and sugar-D moieties; however, their comparable activity suggests that these modifications do not significantly impact anti-TB activity within the spirotetronate polyketide class.

Lobophorin I (**70)**, which has an aldehyde at C-24, demonstrated a twofold reduction in activity (MIC_90_ 3.08 µg mL^–1^) compared to **68**, suggesting that the functional group at C-24 plays a role in anti-TB activity. Moreover, lobophorin F (**71**), which lacks one sugar moiety and contains a methyl unit at C-24, exhibited a sixfold decrease in activity (MIC_90_ 8.01 µg mL^–1^) relative to **68**. The most significant reduction in activity was observed in lobophorin H (**72**), which lacks three sugar moieties, resulting in a complete loss of activity (MIC_90_ > 5.29 µg mL^–1^).

### Anthraquinone

**Fig. 16:**
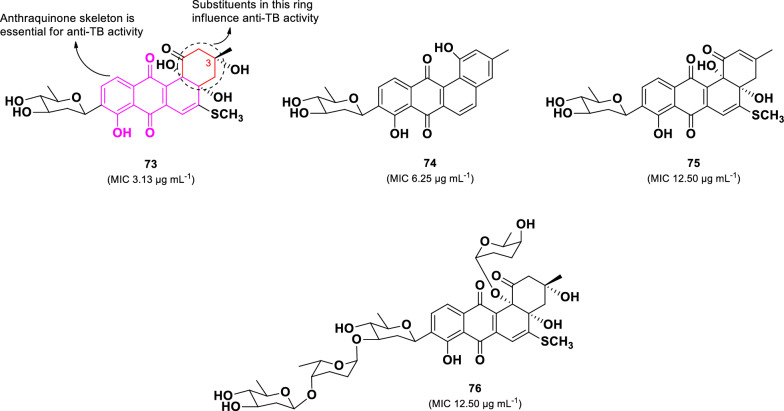
Chemical structures of C-glycosylated benz[a]anthraquinones (**73**–**76**)

A bioactivity-guided investigation of the marine-derived *Streptomyces* sp. (BCC45596) collected in Thailand led to the isolation of four C-glycosylated benz[a]anthraquinone analogs (**73**–**76)** (Fig. 16) [104]**.** Among them, urdamycinone E (**73**) exhibited the highest anti-TB activity against *M. tuberculosis* (H37Ra), with an MIC of 3.13 µg mL^–1^, followed by dehydroxyaquayamycin (**74**, 6.25 µg mL^–1^), urdamycinone G (**75**, MIC 12.50 µg mL^–1^), and urdamycin E (**4**, MIC 12.50 µg mL^–1^). Despite their structural similarities, compounds **75** and **76** demonstrated significantly lower anti-TB activity than compound **73**. The absence of the hydroxyl group at C-3 in **75** or the addition of three sugar units in **76** led to a fourfold decrease in activity. Moreover, compound **74** exhibited greater activity than **75**, suggesting that modifications in the eastern part of the molecule, particularly the formation of a hydroxylbenzene moiety, play a crucial role in enhancing anti-TB activity.

### Diketopiperazine

**Fig. 17:**
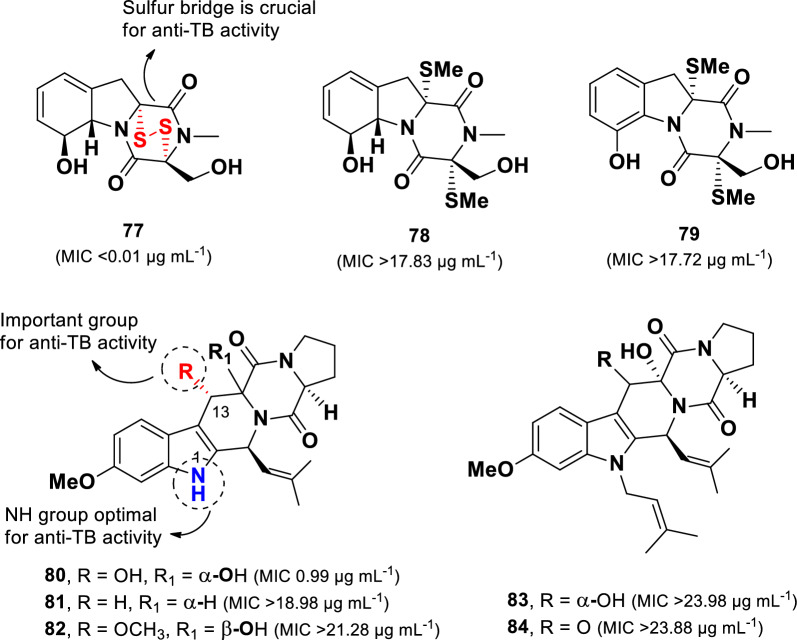
Chemical structures of diketopiperazines (**77**–**84**)

A series of diketopiperazine compounds (**77**–**84**) (Fig. 17) were isolated from the deep-sea-derived fungus *Aspergillus* sp. (SCSIO Ind09F01) collected from sediments in the Indian Ocean [105]. Among them, the sulfur-containing diketopiperazine, gliotoxin (**77)**, exhibited the most potent anti-TB activity (MIC 0.01 µg mL^–1^) against *M. tuberculosis* (H37Ra), surpassing the efficacy of isoniazid (positive control, MIC 0.28 µg mL). In contrast, structurally related sulfur-containing compounds **78** and **79** (MICs > 17.72 µg mL^–1^), displayed no activity, indicating that the presence of a sulfur bridge in gliotoxin **77** is a critical determinant of its anti-TB potency.

Compounds **80**–**84** belong to the fumitremorgin class of diketopiperazines. Structural comparisons within this group indicate that anti-TB activity is influenced by the nature of the nitrogen substituent on the indole ring and the presence of a hydroxyl group at the C-13 position. Among them, 12,13-dihydroxy-fumitremorgin C (**80)** was the only compound that showed anti-TB activity (MIC 0.99 µg mL^–1^). Prenylation at N-1, as observed in **83** and **84** (MICs > 23.98 µg mL^–1^), resulted in complete loss of activity. Furthermore, although compounds **81** and **82** were not prenylated, they also exhibited no activity (MICs > 18.98 µg mL^–1^), indicating that the hydroxyl group at C-13 plays a crucial role in anti-TB activity.

### Naphthalene

**Fig. 18:**
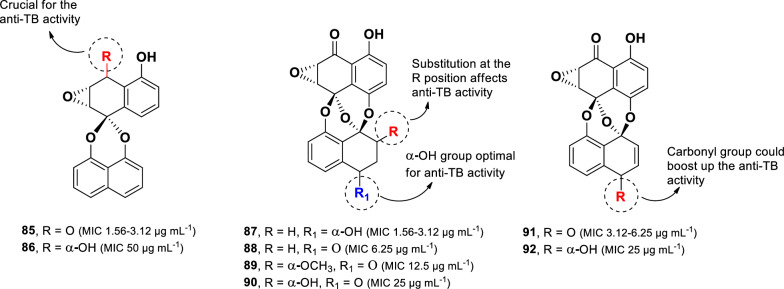
Chemical structures of preussomerins (**85**–**92**)

The bioassay-guided fractionation approach of the fungus *Microsphaeropsis* sp. (strain BCC-3050), isolated from the Thai lichen *Dirinaria applanata* led to the identification of eight preussomerin derivatives (**85**–**92**) (Fig. 18) [106]. Preussomerins are a class of compounds characterized by two naphthalene units connected via oxygen atoms, forming a bis-spiroacetal system [107]. All isolated compounds were screened for their anti-TB activity against *M. tuberculosis* H37Ra. Among them, deoxypreussomerin A (**85**) and preussomerin F (**87**) exhibited the highest activity, with MIC values of 1.56–3.12 µg mL^–1^ and 3.12 µg mL^–1^, respectively. In contrast, bipendensin (**86**, MIC 50 µg mL^–1^), a compound closely related to **85,** differing only in the presence of a hydroxyl instead of a carbonyl group at C-1, showed a great reduction in activity. This suggests that the carbonyl group in 85 is crucial for anti-TB activity. Furthermore, structural modifications at C-2′ and C-3′ influenced activity. The introduction of an unsaturated bond at C-2′, as observed in preussomerin E, led to an eightfold reduction in activity (**92**, MIC 25 µg mL^–1^) compared to **87**. However, when a carbonyl group was present at C-1′, the effect of the unsaturated bond at C-2′ was less pronounced, as observed in preussomerin H (**88**, 6.25 µg mL^–1^) and preussomerin G (**91**, MIC 3.12–6.25 µg mL^–1^). Moreover, modifications at C-3′ also impacted activity. The introduction of a methoxy or hydroxyl group at this position, as seen in preussomerin I (**89**, MIC 12.5 µg mL^–1^) and 3′-*O*-demethylpreussomerin I (**90**, 25 µg mL^–1^), resulted in a twofold and four-fold drop-in activity, respectively.

### Peptide

**Fig. 19:**
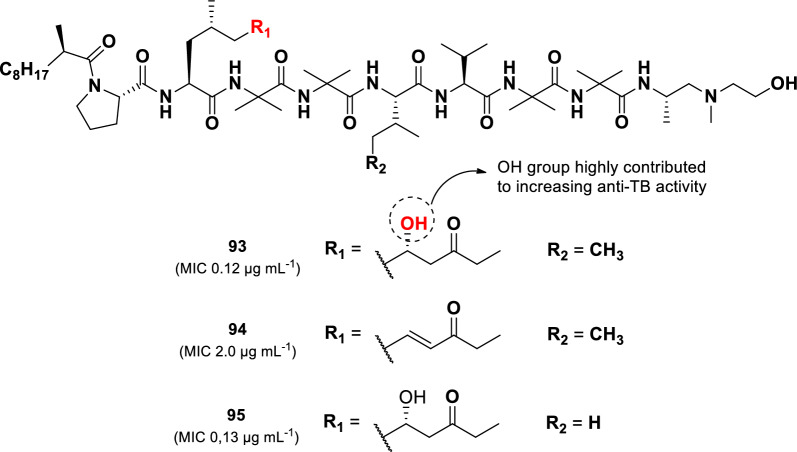
Chemical structures of aminolipopeptides (**93**–**95**)

Another compound with substantial anti-TB activity is produced by the fungus *Glioderma* sp. 05FI48, which was isolated from an unidentified marine sponge [108]. Three aminolipopeptides, trichoderins A (**93**), A1 (**94**), and B (**95**) (Fig. 19), were derived from this strain. They displayed potent activity against *M. tuberculosis* H37Rv under both aerobic and hypoxic conditions, with MIC values of 0.12 µg mL^–1^, 2.0 µg mL^–1^, and 0.13 µg mL^–1^, respectively. Although trichoderins (**93–95**) showed slightly lower in aerobic conditions compared to the positive control isoniazid (MIC 0.05 µg mL^–1^), they were significantly more active under hypoxic conditions, where isoniazid lost effectiveness (MIC > 100 µg mL^–1^). Similar results were observed for activity tests against *M. smegmatis* and *M. bovis* BCG.

Structural variations among these compounds influenced their potency. Compound **94**, which contains an AMOD moiety (2-amino-4-methyl-8-oxodec-6-enoic acid) instead of the AHMOD moiety (2-amino-6-hydroxy-4-methyl-8-oxodecanoic acid) in **93**, showed lower activity. In Contrast, substituting the isoleucine in **93** with valine in **95** did not affect activity, suggesting that the AHMOD moiety plays a crucial role in the anti-TB activity of trichoderins. In the synthetic study of trichoderin A, the stereochemistry of the C-6 in the AHMOD group of compound **93** was confirmed to have the (*R*)-configuration, rather than the previously assigned (*S*)-configuration [109]. Furthermore, the synthetic version of trichoderin A and its C-6 AHMOD epimer exhibited lower anti-TB activity (*M. tuberculosis* H37Ra) than the natural product. This discrepancy is likely due to differences in testing conditions, the synthetic compound was tested as a free base, whereas the natural product was assessed as a salt [109].

Compounds with potent MIC value (< 5 µg mL^–1^) were summarized in Table 1.

**Table 1:** Anti-TB natural product with potent activity against M. tuberculosis (MIC values < 5 µg mL^–1^)

Compound	Species	MIC (µg mL^–1^)	related SAR
Manzamine E (**1**)	*Acanthostrongylophora* sp.	3.80	Manzamine core
6-hydroxymanzamine E (**2**)	*Acanthostrongylophora* sp.	0.40	Hydroxylation C-6 of β-carboline unit
Manzamine F (**3**)	*Acanthostrongylophora* sp.	2.60	Hydroxylation C-8 of β-carboline unit
Manzamine A (**4**)	*Acanthostrongylophora* sp.	1.50	Manzamine core
8-hydroxymanzamine A (**5**)	*Acanthostrongylophora* sp.	0.90	Hydroxylation C-8 of β-carboline unit
Manzamine A-*N*-oxide (**6**)	*Acanthostrongylophora* sp.	3.90	Manzamine core
Neo-kauluamine (**7**)	*Acanthostrongylophora* sp.	1.90	Manzamine core
*ent*-8-hydroxymanzamine A (**8**)	*Acanthostrongylophora* sp.	3.10	Manzamine core
Manzamine J (**9**)	*Acanthostrongylophora* sp.	1.70	Manzamine core
6-deoxy-manzamine X (**10**)	*Acanthostrongylophora* sp.	1.80	Manzamine core
Ircinol A (**11**)	*Acanthostrongylophora* sp.	1.90	Replacing β-carboline with hydroxymethyl
Batzelladine L (**12**)	*Monanchora unguifera*	1.70	Long alkyl chain at c-27; unsaturated bond at c21/c22
Batzelladine N (**13**)	*Monanchora unguifera*	3.20	Short alkyl chain at c-27; double bond c21/c22
Denigrin C (**20**)	*Dendrilla nigra*	4.00	Modification of the pyrrole and hydroxyphenyl rings to form cyclohexenone ring
Ascididemin (**23**)	*Didemnum* sp.	0.10*	Iminoquinone moiety; pyridine ring
Kuanoniamine A (**24**)	*Lissoclinum notti*	3.10*	Iminoquinone moiety; thiazole ring
Heteronemin (**32**)	*Brachiaster* sp.	1.47*	Hydroxyl unit attached at C-12; acetoxy moiety attached at C-19
Heteronemin acetate (**33**)	*Brachiaster* sp.	3.18*	Acetoxy moiety attached at C-12 and C-19
12-deacetoxyscalarin-19-acetate (**34**)	*Brachiaster* sp.	1.71*	Acetoxy moiety attached at C-19
Decarine (**38**)	*Zanthoxylum capense*	1.60	Hydroxyl unit attached at C-8
2′-nortiliacorinine (**45**)	*Tiliacora triandra*	3.10	Secondary amine at N-2′
Tiliacorine (**46**)	*Tiliacora triandra*	3.10	Tertiary amine at N-2′; α-orientation of H-1
Globospiramine (**49**)	*Voacanga globosa*	4.00	Hydroxylation at C-3
Isogoyazensolide (**53**)	*Camchaya calcarea*	1.50	Oxomethylene at C-4; methacyloxy at C-8; α-hydroxyl at C-5
Isocentratherin (**54**)	*Camchaya calcarea*	3.10	Oxomethylene at C-4; angeloyloxy at C-8; α-hydroxyl at C-5
5-*epi*-isogoyazensolide (**55**)	*Camchaya calcarea*	3.10	Oxomethylene at C-4; methacyloxy at C-8; β-hydroxyl at C-5
5-*epi*-isocentratherin (**56**)	*Camchaya calcarea*	3.10	Oxomethylene at C-4; angeloyloxy at C-8; β-hydroxyl at C-5
Goyazensolide (**57**)	*Camchaya calcarea*	3.10	Hydroxymethyl at C-4; methacyloxy at C-8
Tratherin (**58**)	*Camchaya calcarea*	3.10	Hydroxymethyl at C-4; angeloyloxy at C-8
Plumbagin (**60**)	*Diospyros anisandra*	1.56	Carbonyl at C-1; double bond C-2/C-3
Maritinone (**63**)	*Diospyros anisandra*	3.13	Dimer naphtoquinone
3,3′-biplumbagin (**64**)	*Diospyros anisandra*	3.13	Dimer naphtoquinone
Lobophorin B (**68**)	*Streptomyces* sp.	1.54* (MIC_90_)	Three sugar unit attached at C-9; hydroxylmethyl at C-22
Lobophorin C (**69**)	*Streptomyces* sp.	1.62* (MIC_90_)	Three sugar unit attached at C-9; hydroxylmethyl at C-22
Lobophorin I (**70**)	*Streptomyces* sp.	3.08* (MIC_90_)	Three sugar unit attached at C-9; aldehyde at C-22
Urdamycinone E (**73**)	*Streptomyces* sp.	3.13	Hydroxylation at C-3; a sugar moeity at C-9;
Gliotoxin (**77**)	*Aspergillus* sp.	< 0.01*	Sulphur-bridge
12,13-dihydroxy-fumitremorgin C (**80**)	*Aspergillus* sp.	0.99*	Secondary amine at N-1; hydroxylation at C-13
Deoxypreussomerin A (**85**)	*Microsphaeropsis* sp.	1.56–3.12	Carbonyl at C-1
Preussomerin F (**87**)	*Microsphaeropsis* sp.	3.12	Hydroxylation at C-1′; unsaturated bond at C2′
Preussomerin G (**91**)	*Microsphaeropsis* sp.	3.12–6.25	Carbonyl at C-1′
Trichoderin A (**93**)	*Trichoderma* sp.	0.12	2-amino-6-hydroxy-4-methyl-8-oxodecanoic acid
Trichoderin A1 (**94**)	*Trichoderma* sp.	2.00	2-amino-4-methyl-8-oxodec-6-enoic acid
Trichoderin B (**95**)	*Trichoderma* sp.	0.13	2-amino-6-hydroxy-4-methyl-8-oxodecanoic acid

*converted from µM to µg mL^–1^

While the molecules that regulatory approved for commercially were protemanid (MIC: 0.015–0.531 µg mL^–1^), moxiflaxacin (MIC: 0.125—0.5 µM, bedaquiline (MIC: 0.06 µg mL^–1^), rifampentin (MIC: 0.125–0.25 µg mL^–1^), and delamanid (MIC: 0.403 μM) [13, 110–112].

**Fig. 20:**
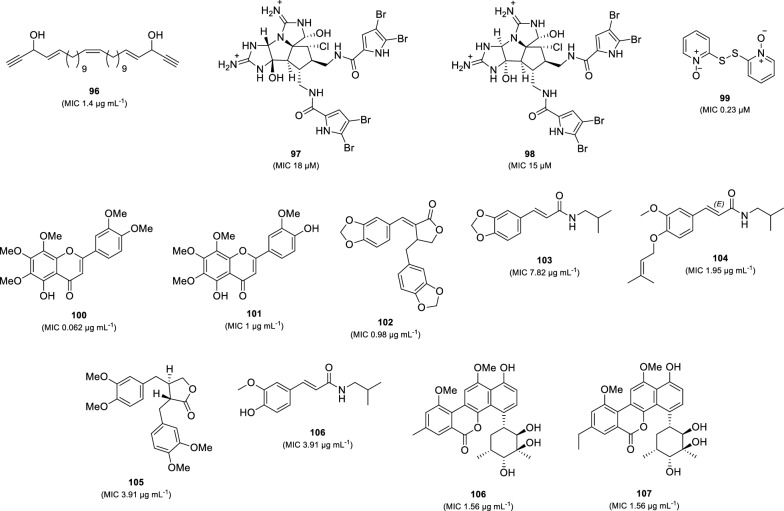
Chemical structure of the newest potent natural anti-TB compounds

Recently, several natural products with potent anti-TB activity (MIC less than 20 µM or 20 µg mL^–1^) were reported from marine organisms, plants, and microorganisms (Fig. 20). A linear acetylene, duryne (**96**), was isolated from the Solomon Islands marine sponge *Petrosia* sp. This compound exhibited inhibitory activity against *M. tuberculosis* (H37Rv) with an MIC value of 1.4 µg mL^–1^ [113]. Two anti-TB pyrrole alkaloids, axinellamines A (**97**) and B (**98**), were isolated through chemical investigation using bioassay-guided fractions of an Australian marine sponge (order Haplosclerida) [114]. Both compounds inhibited *M. tuberculosis* (H37Rv) with MIC_90_ values of 18 µM and 15 µM, respectively, and were not toxic when tested at 25 µM to mamalian cells. Dipyrithione (**99**), a sulfur-containing pyridine, was isolated from the stem of *Marsypopetalum modestum* (Champasak Province, Laos) [115]. Compound **99** showed potent anti-TB activity against *M. tuberculosis* (H37Rv) with an MIC value of 0.23 µM. However, this compound also exhibited cytotoxicity against HepG2 cells (IC_50_ 0.8 µM) and HCT116 cells (IC_50_ 4.1 µM). Investigation on the aerial part of *Galatella grimmii* (Khorasan-Razavi Province, Iran) led to the isolation of two anti-TB flavonoids, 5-demethylnobiletin (**100**) and 8-methoxycirsilineol (**101**) [116]. Compounds **100** and **101** exhibited strong anti-*M. tuberculosis* H37Rv activity with MICs of 0.062 and 1 µg mL^–1^. Furthermore, 5-demethylnobiletin (**100**) also inhibited all tested strains of drug-resistant *M. tuberculosis* with MICs ranging from 8 to 16 µg mL^–1^. ( ±) savinin (**102**, MIC 0.98 µg mL^–1^), *trans*-fagaramide (**103**, MIC 7.82 µg mL^–1^), 4-(isoprenyloxy)-3-methoxy-3,4-deoxymethylenedioxyfagaramide (**104**, MIC 1.95 µg mL^–1^), maculatin (**105**, MIC 3.91 µg mL^–1^), and (*E*)-3-(4-hydroxy-3-methoxyphenyl)-N-isobutylacrylamide (**106**, MIC 3.91 µg mL^–1^), were discovered as potent anti-TB compounds against *M. tuberculosis* (H37Rv) from the *Zanthoxylum leprieurii* (root bark) collected at Central Uganda [117]. Moreover, compound **106** displayed moderate activity against a strain of MDR-TB with an MIC value of 31.25 µg mL^–1^. Another potent anti-TB compounds were reported from *Streptomyces* sp. MS751 [118]. Two C-glycoside polyketides isolated from this strain, chrysomycins B (**107**) and C (**108**), exhibited strong anti-TB activity against *M. tuberculosis* (H37Rv) with an MIC value of 1.56 µg mL^–1^. Moreover, these compounds also showed activity against several MDR-Tb strains (MICs 1.56–3.23 56 µg mL^–1^).

## Correlation of anti-TB activity and cytotoxicity against cell culture

In the early stages of drug discovery, evaluating biological activity and cytotoxic profiles is crucial for identifying promising candidates [119, 120]. Ideally, compounds that show potent biological activity with minimal or no cytotoxicity to host cells serve as attractive scaffolds for drug research [81, 121].

This section highlights the relationship between anti-TB activity and cytotoxicity in several potent compounds, which may aid in selecting potential scaffolds for TB drug research. In addition, SAR analysis of the selected compounds was conducted to identify key factors influencing their anti-TB activity. A detailed description of the analytical can be found in the Supplementary Materials.

**Fig. 21:**
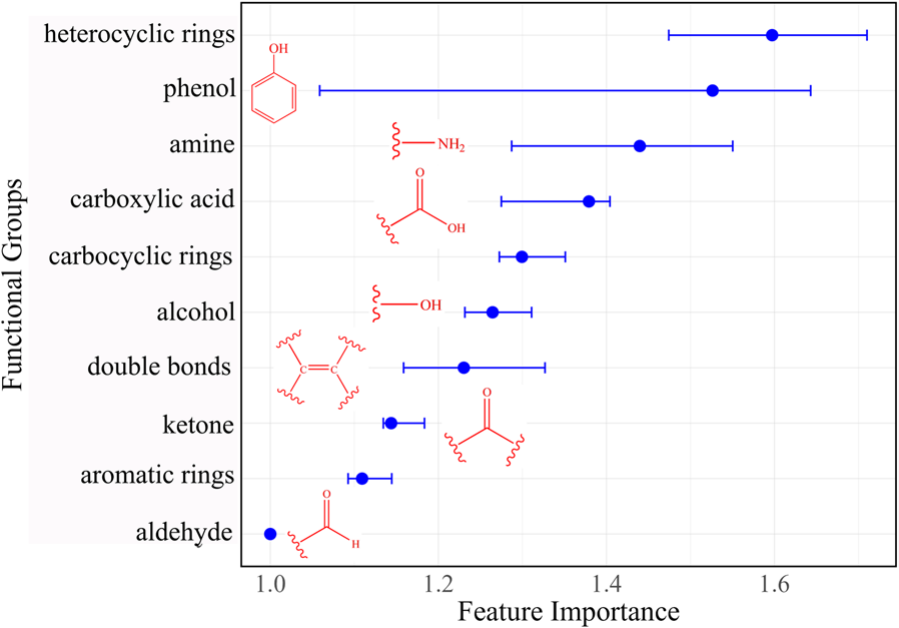
Permutation importance of molecular descriptors, specifically ring system, ring counts and functional groups, to MIC. The impact of each descriptor is represented using mean square error (MSE) as the loss function. A higher feature importance value indicatesa greater impact of the descriptor on the MIC

The impact of specific compound features, such as ring systems and functional group composition, on minimum inhibitory concentration (MIC) values is illustrated in Fig. 21**.** The analysis revealed that ring systems significantly affect MIC values. Compounds containing heterocyclic rings were generally associated with higher MIC values, indicating reduced anti-TB potency. Conversely, the incorporation of aromatic rings into the ring structures led to an increase in MIC values, suggesting lower activity. Furthermore, the presence of phenolic moieties and amine groups in the correlated with a more pronounced reduction in MIC values, emphasising their role in improving anti-TB activity. These findings provide valuable insights into the structural features that contribute to the efficacy of anti-TB compounds and may guide future drug development efforts.

6-Hydroxymanzamine E (**2**, MIC 0.4 µg mL^–1^) and 8-hydroxymanzamine A (**5**, MIC 0.9 µg mL^–1^) are two potent anti-TB compounds from marine organisms. Structural comparisons indicate that hydroxylation at positions C-6 and C-8 in the β-carboline ring is crucial for anti-TB activity. Compounds **4** and **5** exhibited significant cytotoxicity against the normal Vero cell, with MIC values of 1.1 µg mL^–1^ and 1.2 µg mL^–1^, respectively [58, 59]. The cytotoxicity of manzamines appears to be influenced by the modification of the eight-membered ring, as evidenced by the inactivity of **2** and other derivatives (**1**, **3**, **7**, **9–11**) that exhibit structural variations in this region at a concentration of 4.7 µg mL^–1^. Moreover, (-)-ircinol A (**11**, MIC 1.9 µg mL^–1^) displayed activity comparable to that of compound **4,** with no detectable cytotoxicity at 4.7 µg mL^–1^. Notably, the stereochemistry of **11** has been identified as unfavorable for anti-TB activity based on comparisons of compounds **5** and **8**. Therefore, further investigation into its enantiomer may be of interest.

Another type of alkaloid derived from marine organisms, with significant anti-TB activity (**23,** MIC 0.10 µg mL^–1^), is ascididemin of the pyridoacridine alkaloid class. SAR analysis suggests that the complete iminoquinone moiety is crucial for its activity. However, ascididemin also exhibited strong cytotoxicity against Vero and P388 cell lines, with IC_50_ values of < 0.14 µM and 0.4 µM, respectively [66]. The cytotoxic effects of this compound may be attributed to its large planar aromatic structure with positively charged nitrogen atoms, which can contribute to telomerase inhibition by stabilizing G-quadruplex DNA [122]. Further investigation into the synthetic compound 4-substituted-pyrido[2,3,4-*kl*]acridin-6-one led to the identification of a potent analog, 4-ethylthiopyrido[2,3,4-kl]acridin-6-one (**31**, MIC 0.10 µg mL^–1^), which displays anti-TB activity similar to that of the parent compound (**23**) while exhibiting significantly reduced cytotoxicity (Vero: IC_50_ 6.8 µM; P388: IC_50_ 6.8 µM) [66]. Additionally, compound **31** demonstrated potent activity against several strains of single-drug-resistant *M. tuberculosis,* with MIC values ranging from 0.2 to 0.4 µM. Moreover, amino analogs such as ethyl-pyrazine-carboxamide and ethyl-pyrazine-carboxylate exhibited potent anti-TB activity (MIC 0.79 µg mL^–1^) with negligible cytotoxicity against Vero and P388 cell lines (IC_50_ > 25 µM) [66].

Beyond the alkaloid class, marine organisms also produce potent anti-TB from scalarane-type sesterterpene, with heteronemin (**32**) and 12-deacetoxyscalarin-19-acetate (**34**) emerging as the most active representatives of this group. The anti-TB activity of these compounds is influenced by two structural features: the presence of acetoxy at C-19 and the nature of the substituent at C-12. Notably, compound **34** exhibited no cytotoxicity against various cancer cell lines (HT-29, MCF-7, KB, and HeLA) when tested at 10 µM, whereas compound **32** demonstrated strong cytotoxicity, with IC_50_ values ranging from 0.29 to 0.45 µM [68].

Structural modifications at C-12 further impact biological activity, as seen in compounds **35** (hydroxyl at C-12) and **36** (acetoxy at C-12), both of which displayed cytotoxicity against the tested cancer cell lines (IC_50_: 0.26–1.99 µM). The semi-synthetic derivatives bearing a ketone moiety at C-12 (**37)** showed an enhanced anti-TB activity (MIC 0.11 µg mL^–1^). However, it also displayed increased cytotoxicity in normal human oral fibroblasts (IC_50_ 0.91 µM) compared to its parent compound, **32** (IC_50_ 5.25 µM). These findings are consistent with previous studies indicating that substituent modifications at C-12 play a crucial role in both anti-TB activity and cytotoxicity. Based on these results, 6-hydroxymanzamine E (**2**), ircinol A (**11**), the thioethyl analog of ascididemin (**31**), and 12-deacetoxyscalarin 19-acetate (**34**) represent promising scaffolds for future studies in the development of novel anti-TB agents.

Several chemical constituents display potent anti-TB activity with low cytotoxicity, one of which is the benzophenanthridine alkaloid decarine **(38**, MIC 1.6 μg mL^–1^; THP-1: IC_50_ 66 µg mL^–1^) [84]. The position and type of substituent on ring A significantly influence the anti-TB activity within this class. Specifically, replacing the hydroxyl group at C-8 with a methoxy group results in the loss of activity. A structural comparison between compounds **40** and **42** reveals that cytotoxicity (THP-1) is closely related to the position of the methoxy group on ring A. Oxygenation at C-8 and C-9, as observed in **40** (IC_50_ 1.7 µg mL^–1^), increases the cytotoxicity approximately 50-fold compared to oxygenation at C-7 and C-8 in compound **42** (IC_50_ 57.1 µg mL^–1^). Additionally, compounds lacking oxygenation at C-9 (**39, 41**–**44**) exhibited weak cytotoxicity, with IC_50_ values ranging from 43.7 to 94.5 µg mL^–1^.

Three derived bisbenzoquinolines (**45**–**47)** and a synthetic derivative (**48**) demonstrated remarkable anti-TB activity, with MIC values ranging from 3.1 to 6.2 µg mL^–1^. The introduction of a bromine atom in the bisbenzyl moiety enhanced activity, whereas the methylation at N-2′ resulted in a twofold reduction in potency. Cytotoxicity assessment in normal MRC-5 cells revealed that compounds **45–47** exhibited cytotoxicity (IC_50_: 3.13–3.87 µg mL^–1^) [86], while the synthetic derivative **48** displayed less cytotoxicity with (IC_50_: 20.0 µg mL^–1^). Notably, the addition of a bromine atom to the bisbenzyl unit increased anti-TB activity while simultaneously reducing cytotoxicity twofold.

Beyond alkaloids, sesquiterpene lactones represent another potent anti-TB class derived from plants. Seven compounds in this group (**53–59**) exhibited high anti-TB activity, with MIC values ranging from 3.1 to 6.2 µg mL^–1^. The primary structural components critical for anti-TB properties include the lactone moiety and the heterocyclic eight-membered ring system. Compounds **53**, **54**, **57–59** demonstrated strong cytotoxicity against Vero cells, with IC_50_ values ranging from 0.2 to 5.8 µg mL^–1^ [90]. Interestingly, compound **55** (IC_50_: 12.6 µg mL^–1^), which features a β-oriented hydroxyl group at C-5, exhibited significantly lower cytotoxicity compared to its epimer **53** (IC_50_: 0.2 µg mL^–1^). Additionally, the substituents at C-8 played a role in cytotoxicity variation, as observed in the comparison between compounds **57** and **58**.

Two plant-derived dimeric naphthoquinone, maritinone (**63)** and 3,3′-biplumbagin (**64**), exhibited promising anti-TB properties (MIC 3.13 µg mL^–1^) with low cytotoxicity against Vero cells (IC_50_:232.68 and 607.57 µg mL^–1^, respectively) [91]. Structural analysis suggests that dimerization is critical for both anti-TB activity and cytotoxicity. In contrast, the monomeric analog **60** (MIC 1.56 µg mL^–1^) displayed twofold higher anti-TB potency than compounds **63** and **64** but exhibited significant cytotoxicity (IC_50_: 0.15–3.73 µg mL^–1^). Therefore, compounds such as decarine (**38**), 13′-bromo-tiliacorinine (**48**), 5-epi-isogoyazensolide (**55**), maritinone (**63),** and 3,3′-biplumbagin (**64**) could serve as promising frameworks for further anti-TB drug development.

Among bacterial-derived compounds, three spirotetronate polyketides, lobophorins B (**68**, MIC_90_ 1.3 µg mL^–1^), C (**69**, MIC_90_ 1.4 µg mL^–1^), and I (**70**, MIC_90_ 2.6 µg mL^–1^), showed potent anti-TB activity. The sugar unit and functional group at C-24 are crucial for their activity. Unfortunately, the two most potent compounds, **68** and **69**, exhibited significant cytotoxicity against lymphoblastoid cells (CEM-TART), with LD_50_ values of 1.6 and 1.7 µM, respectively [102]. Moreover, compound **71 (**LD_50_ 0.3 µM), which contains only two sugar groups, exhibited even higher cytotoxicity than compounds **68** and **69,** which contain three sugar groups. Interestingly, the presence of an aldehyde group at C-24 in compound **70 (**LD_50_ 8.6 µM) decreased the cytotoxicity fivefold compared to the secondary alcohol in **68**. These findings suggest that substituents at C-24 play a key role in cytotoxicity modulation.

The fungal-derived compound 12,13-dihydroxy-fumitremorgin C (**80)** displays potent anti-TB activity (MIC 1.29 µg mL^–1^) with no detectable cytotoxicity (IC_50_ > 50 μM) against A549, Huh-7, and K562 cancer cell lines [105]. The key structural features contributing to its anti-TB activity are the secondary amine in the indole ring and the hydroxyl group at C-33. Notably, similar compounds (**81**–**84**) were also inactive against these cancer cell lines at a concentration of 50 μM.

Another promising fungal-derived anti-TB compound is deoxypreussomerin A (**85**, MIC 1.56–3.12 µg mL^–1^). The functional group at the C-1 position significantly influences the anti-TB activity, with the carbonyl group being preferred over the hydroxyl group. Importantly, deoxypreussomerin A (Vero cells: IC_50_ 21.8 µg mL^–1^), which contains only two oxygen bridges connecting the naphthalenes, demonstrated much lower cytotoxicity than structurally related compounds with three oxygen bridges (**87–92**, IC_50_ ≤ 1 µg mL^–1^) [106]. These findings highlight lobophorin I (**70**), 12,13-dihydroxy-fumitremorgin C (**80**), and deoxypreussomerin A (**85**) as promising scaffolds for further anti-TB drug development.

Additionally, in silico experiments were performed to investigate the interactions of the selected compounds with ten key *Mycobacterium tuberculosis* (Mtb) proteins involved in essential metabolic pathways (Table S1). These pathways, include cell wall biosynthesis (arabinogalactan, mycolic acid, and peptidoglycan), fatty acid biosynthesis, protein synthesis, signal transduction, cofactor biosynthesis (Coenzyme A and biotin), aspartate biosynthesis, and substrate recognition and unfolding. Detailed docking and re-ranking protocols are provided in the Supplementary Materials.

**Fig. 22:**
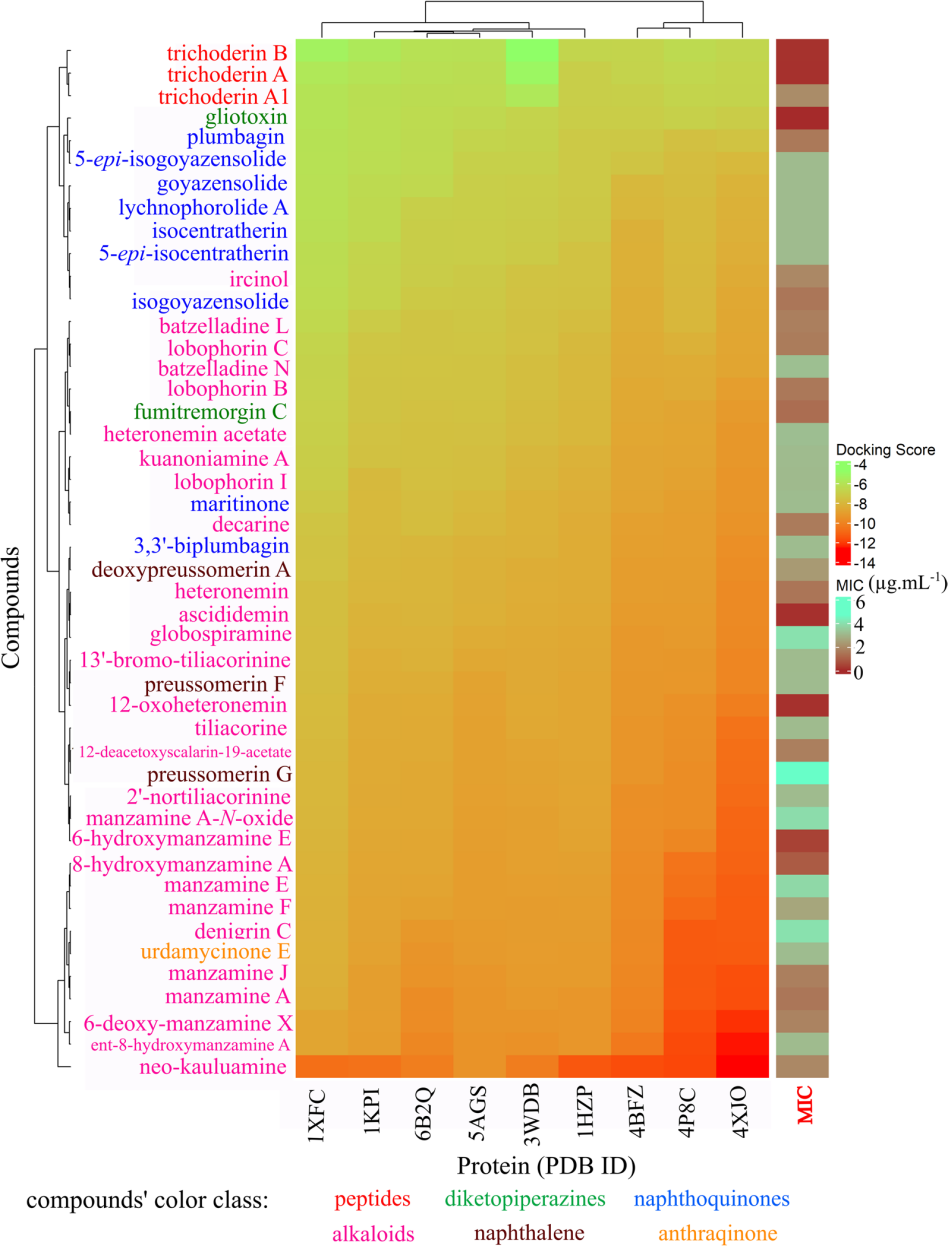
The ComplexHeatmap for re-ranking docking score after XGBoost model. PDB ID 1XFC (Alanine racemase alr), 6U7A (Aspartate aminotransferase aspAT), 1KPI (Mycolic acid cyclopropane synthase CmaA2), 6B2Q (Protein kinase A PknA), 5AGS (leucyl-tRNA synthase LeuRS), 3WDB (N-terminal domain of Mycobacterium tuberculosis ClpC1), 1HZP (3-oxoacyl-[acyl-carrier-protein] synthase 3 FabH), 4BFZ (Pantothenate kinase PanK, type 1), 4P8C (Decaprenylphosphoryl-β-d-ribofuranose oxidoreductase DprE1), 4XJO (5′-pyridoxal phosphate (PLP)-dependent aminotransferase (BioA)

Docking analysis revealed that all tested compounds exhibited interactions with the target proteins (Fig. 22). Notably, they demonstrated stronger binding affinities toward 4XJO (cofactor biosynthesis: biotin biosynthesis pathway), 4BFZ (cofactor biosynthesis: Coenzyme A biosynthesis pathway), and 4P8C (cell wall biosynthesis: arabinogalactan biosynthesis). These findings suggest that the compounds could effectively disrupt essential metabolic pathways in Mtb. Among the tested compounds, neo-kauluamine showed the strongest activity, whereas trichoderin B, despite its low MIC, demonstrated the weakest overall effectiveness.

## Conclusion and future perspective

The prevalence of drug-resistant *M. tuberculosis* infections is driven by prolonged and ineffective drug administration, along with worsening environmental conditions that facilitate genetic mutations Exposure to bactericidal antibiotics enables the survival of certain bacterial populations, leading to phenotypic adaptations and genetic mutations that enhance resistance. *M. tuberculosis* exhibits resistance not only to long-established drugs but also to newly developed therapies, necessitating the continuous search for novel anti-TB agents.

To combat *M. tuberculosis* resistance, new drug candidates are being designed to target critical metabolic pathways, including DNA gyrase, cell wall biosynthesis, oxidative phosphorylation, efflux pumps, and intermediate metabolism. These targeted strategies aim to disrupt essential bacterial functions, thereby overcoming resistance mechanisms. While in vitro testing remains the standard for evaluating drug [26], discrepancies between experimental results and clinical outcomes highlight the need for more advanced approaches. Rational drug design and SAR studies play an indispensable role in optimizing drug candidates, ensuring better alignment with therapeutic goals [26].

In this study, 43 natural products demonstrated potent anti-TB activity (MIC < 5 µg mL^–1^). Contributions from marine organisms, plants, and microorganisms were nearly equivalent, with 18, 13, and 12 compounds, respectively. Notably, the most potent anti-TB compounds (MIC < 1 µg mL^–1^) were derived from microorganisms and marine organisms (**2**, **5**, **23**, **77**, **93**, **95**), while plant-derived compounds showed slightly lower anti-TB activity but with reduced cytotoxicity (**38**, **63**, **64**).

One of the key challenges in TB drug discovery is that many potent natural products also exhibit high cytotoxicity, limiting their therapeutic potential. For example, gliotoxin (**77**), ascididemin (**23**), and 8-hydroxymanzamine A (**5**) showed remarkable anti-TB activity (MIC < 1 µg mL^−1^) but were accompanied by strong cytotoxic effects. Structural modification is a rational strategy to overcoming this issue, as observed in the development of rifampicin, rifabutin, and rifapentine from rifamycin. Identifying promising scaffolds with potent anti-TB activity and low cytotoxicity remains a priority for future research.

Based on the correlation between anti-TB activity and cytotoxicity in this study, several natural products appear to be promising scaffolds for future anti-TB drug development. These include marine-derived compounds (**2**, **11**, **31**, **34**), plant-derived (**38**, **48**, **55**, **63**, **64**), and microorganism-derived (**70**, **80**, **85)**. Additionally, since both biological activity and cytotoxicity are crucial in the early stages of drug discovery, simultaneous evaluation of these properties in a single experiment is essential for optimizing lead identification.

Among the prioritized compounds with MIC < 1 µg mL⁻^1^, ascididemin (**23**), trichoderin A (**93**), and trichoderin B (**95**) fulfilled all SwissADME drug-likeness criteria ((http://www.swissadme.ch/index.php)) without violating any parameter (Table S3E, excel file). These criteria include Lipinski’s Rule of Five (predicting oral bioavailability), the Ghose filter (physicochemical properties), Veber rule (molecular flexibility and polarity), Egan rule (oral absorption and blood–brain barrier permeability), and the Muegge filter (drug-like chemical space). Meanwhile, compounds **2**, **5**, and **77** complied with Lipinski and Veber rules, with compounds **2** and **5** also meeting the Egan rule. Therefore, compounds **2**, **5**, **23**, **77**, **93**, and **95** are predicted to possess favorable oral bioavailability. Overall, this study reinforces that natural products remain a rich and valuable source of anti-TB compounds, offering promising candidates for future drug development.

## Supplementary Information

Additional file 1.

Additional file 2.

## Data Availability

All the data in the manuscript are obtained from included references and available upon request.
