# Supplementary material for: Unlocking potent anti-tuberculosis natural products through structure–activity relationship analysis
Source: Nat Prod Bioprospect. 2025 Jul 7;15(1):44. doi: 10.1007/s13659-025-00529-4 (PMC12234934; doi:10.1007/s13659-025-00529-4)
Supplement: Supplementary file 1 — Additional file 1. [file 13659_2025_529_MOESM1_ESM.docx]

**Unlocking Potent Anti-Tuberculosis Natural Products through Structure-Activity Relationship Analysis**

Delfly Booby Abdjul,^1,2*^ Fitri Budiyanto,^1^ Joko Tri Wibowo,^1^ Tutik Murniasih^1^, Siti Irma Rahmawati,^1^ Dwi Wahyu Indriani,^1^ Masteria Yunovilsa Putra^1^ and Asep Bayu^1**^

^1^Research Center for Vaccine and Drugs, Research Organization for Health, National Research and Innovation Agency (BRIN), Jalan Raya Jakarta Bogor KM.46, Cibinong, Bogor, West Java, Indonesia 16911

^2^North Sulawesi Research and Development Agency, Jalan 17 Agustus, Manado, North Sulawesi, Indonesia 95116

corresponding author(s): ^*^ [booby_abdjul@yahoo.com](mailto:booby_abdjul@yahoo.com); [**asep044@brin.go.id](mailto:**asep044@brin.go.id)

1. **Methodology**
2. **Overview of literature collection**

This critical review paper went through literature collection, data sorting, and data processing (Figure S1). Two scientific search engines, Scopus and PubMed, were utilised for data collection. For each search engine, five types of keywords were utilised. *i.e.* antitubercular AND marine, antitubercular AND sponge, antitubercular AND fungi, antitubercular AND bacteria, and antitubercular AND plants. The amount of literature from each search engine is presented in Figure S2. The data was refined to compounds with potent MIC value (< 5 µM).


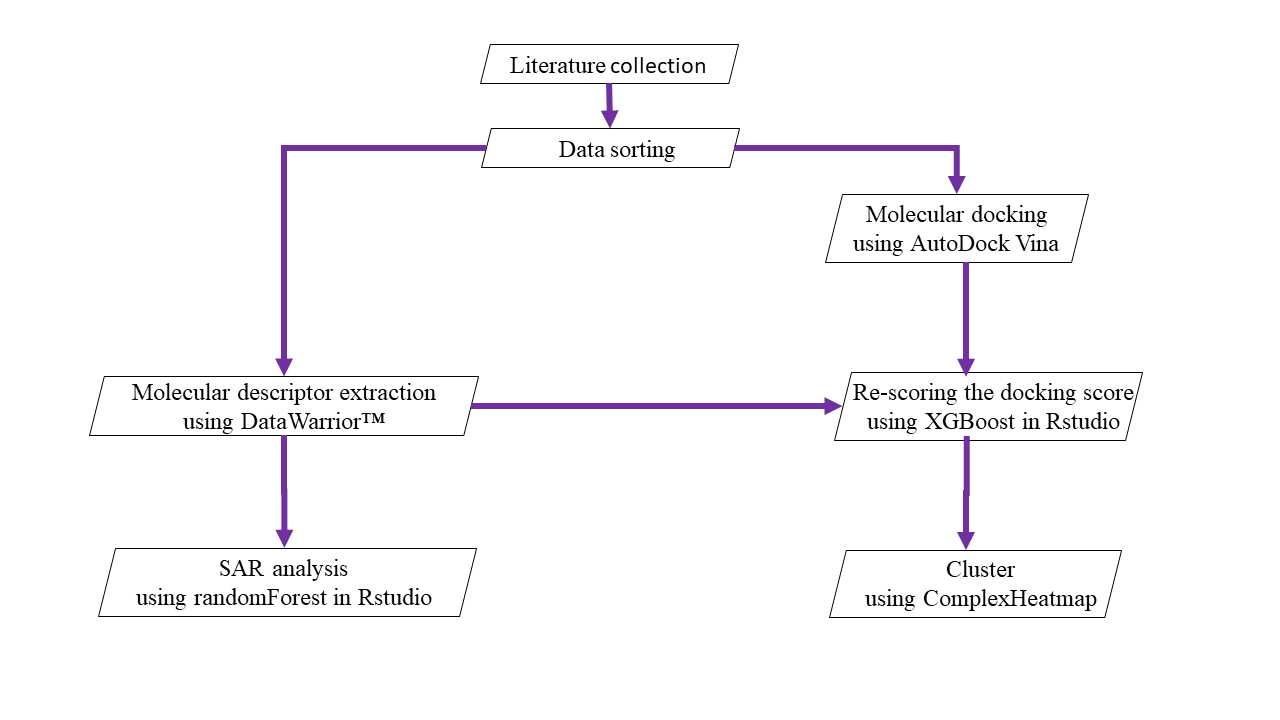


**Figure S1.** General overview of methodology use in this critical review.


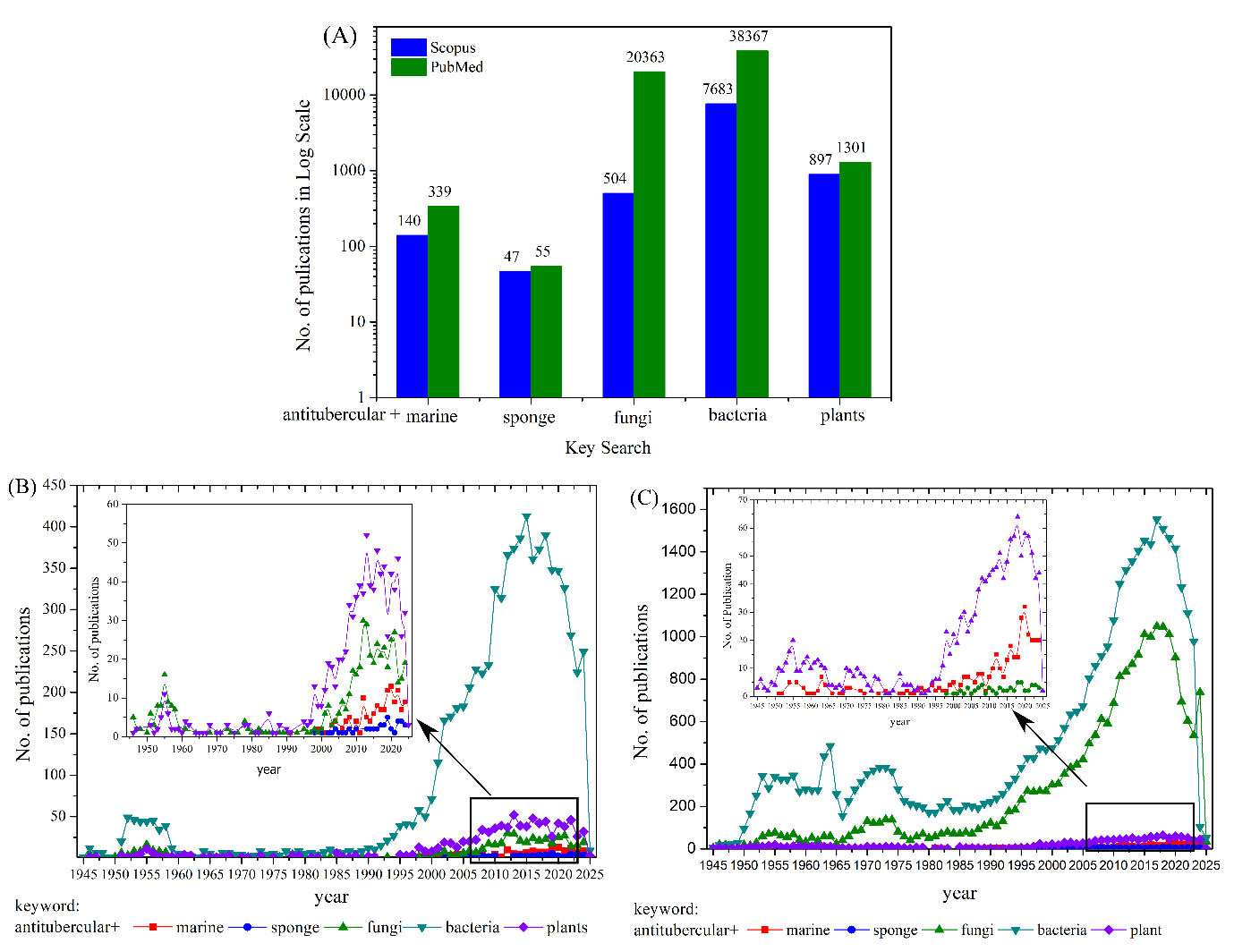


**Figure S2.** Number of publication appear in the literatures’ exploration using proposed keywords: (A) total publication from each search engines, (B) publication in Scopus annum, (C) publication in PubMed annum.

1. **Molecular descriptor extraction using DataWarrior.**

The list of compounds from the data collection step was utilized as input data for molecular descriptor extraction using DataWarrior^1^. The SMILES format of each compound was obtained from the PubChem website (<https://pubchem.ncbi.nlm.nih.gov/>, accessed from Dec 5th-10th, 2024) or constructed with the Marvin JS website (<https://marvinjs-demo.chemaxon.com/latest/demo.html>, accessed from Dec 5^th^-10^th^, 2024). The molecular descriptors extracted from DataWarrior comprises of: druglikeness, ligand efficiency and shape, atom counts, ring counts, functional groups, and 3D properties. The specific functional group was computed from python using RDKit.

1. **RandomForest model for SAR analysis**

The algorithm used in the RandomForest modelling applied bootstrap aggregating (bagging) followed by random feature selection, adopting from Svetnik *et al.* (2003)^2^ and Trinh *et al.* (2022)^3^ with modification. The model was predicted using the RandomForest package in R4.4.1 software. The molecular descriptor from DataWarrior was set as a feature vector, while the MIC was set as the target variable. The input data was prepared as Table S1E in excel file. The dataset underwent cleaning and transformation in the data preparation step, followed by feature encoding to convert categorical features to numeric features. The dataset (D) used here is prepared as follows.

$D=\left\{ \left( X_{i},Y_{i} \right) \right\}_{i=1}^{n}$, (1)

Where $X_{i}$ is molecular descriptors such as rings system and functional groups as feature vectors and $Y_{i}$ is MIC value as the target variable.

Then, to reduce variance and introduce variability in the model, Bootstrap sampling ($D_{b}^{*}=(b-1,2,...,B), B is number of trees)$ was generated. This bootstrap sampling contains 63.2% of the original data.

The tree was constructed for each bootstrap sample and grew as an unpruned decision tree *T_b_*. At each node in the tree, the number of features randomly sampled at each split (*m*_try_) was selected from the total *p* features. The best spilt was selected based on a criterion of Gini Impurity (classification) and Mean Square Error (regression). The Gini Impurity and MSE were calculated as follows.

$Gini\left( t \right)=1-\sum_{k=1}^{K} P_{k}^{2}$, (2)

$MSE\left( t \right)=\frac{1}{N_{t}}\sum_{i=1}^{N_{t}} \left( y_{i}-\bar{y_{t}} \right)^{2}$, (3)

Where $P_{k}$ is the proportion of class *k* in the node *t,* $N_{t}$ is the number of samples in node *t*, and $y_{t}$ is the mean response.

To obtain a prediction dataset $h_{b}\left( Xi \right)$, the feature vector Xi was passed through each tree Tb. Then, the prediction aggregation was computed using classification (using majority voting) and regression (using average) as follows.

$Classification, H(X)=mode\left\{ h_{1}\left( X \right), h_{2}\left( X \right), ..., h_{B}\left( X \right) \right\}$ (4)

$Average, H(X)=\frac{1}{B}\sum_{b=1}^{B} h_{b}\left( X \right)$ (5)

The result was presented as a plot of Feature Importance (loss:MSE) vs molecular descriptor. The permutation importance or feature importance (loss:MSE) reflects the importance of a feature, measured using mean square error (MSE) as the loss function. Thus, higher importance means the feature (molecular descriptor) to have a higher impact on the model’s ability to predict the MIC.

1. **Molecular docking using Autodock Vina**

The protein target for the docking was selected according to several inhibition pathways of *Mycobacterium tuberculosis*. Ten (10) proteins with different PDB IDs were selected for this *in silico* study (Table S1). The 3D structures of these proteins were obtained from the RCSB Protein Data Bank (PDB) (<https://www.rcsb.org>, accessed on December 14^th^, 2024). The 3D configuration of each compounds was obtained from PubChem website (<https://pubchem.ncbi.nlm.nih.gov/>, accessed from Dec 5th-10th, 2024) or draw on Marvin JS website (<https://marvinjs-demo.chemaxon.com/latest/demo.html>, accessed from Dec 5^th^-10^th^, 2024). The protein and compounds were prepared by using AutoDock tools1.5.7 before the docking procedure. The protein preparation includes removing water molecules, adding polar hydrogen, and applying Kollman charges to ensure precise docking accuracy ^4^. Subsequently, molecular docking was executed using Autodock Vina1.1.2. To validate the docking experiments, re-docking of native ligands was performed.

1. **Re-ranking docking score using XGBoost**

The Vina docking score was re-ranking using the machine learning XGBoost package in R4.4.1 software, adopting the procedure from Bande and Baday (2023)^5^ and Yang and Zhang (2022)^6^. The model was built by combining the docking score from Vina with the molecular descriptor as prepared in Table S2E (excel file). The molecular descriptor and PDB ID were set as features (independent variable), while Vina’s docking score was set as target variables (dependent variables). Then, the data was split into a training set (80% of the data) and a validation set (20% of the data). Those data were then transformed into ‘DMatrix’ for gradient boosting. Further, the data was trained using the XGBoost package to predict the docking score. The re-ranked value of the docking score is presented in Table S2.

1. **Cluster of re-ranked docking score**

The re-ranked docking score was presented in a heatmap using the ComplexHeatmap package in R4.4.1 software.

1. **Code for functional group extraction using RDKit in python**

**import** pandas **as** pd

**from** rdkit **import** Chem

*# Function to count functional groups*

**def** count_functional_groups(**smiles**)**:**

    mol **=** Chem**.**MolFromSmiles(smiles)

**if** mol**:**

        functional_groups **=** {

            "Alcohol"**:** 0**,**

            "Aldehyde"**:** 0**,**

            "Carboxylic_Acid"**:** 0**,**

            "Amine"**:** 0**,**

            "Phenol"**:** 0**,**

            "Ketone"**:** 0**,**

            "Ether"**:** 0**,**

        }

        smi **=** Chem**.**MolToSmiles(mol)

*# Count occurrences of functional groups*

        functional_groups["Alcohol"] **=** smi**.**count("O") **if** "O" **in** smi **else** 0

        functional_groups["Aldehyde"] **=** smi**.**count("C=O") **if** "C=O" **in** smi **else** 0

        functional_groups["Carboxylic_Acid"] **=** smi**.**count("C(=O)O") **if** "C(=O)O" **in** smi **else** 0

        functional_groups["Amine"] **=** smi**.**count("N") **if** "N" **in** smi **else** 0

        functional_groups["Phenol"] **=** smi**.**count("c1cc") **+** smi**.**count("O") **if** "c1cc" **in** smi **and** "O" **in** smi **else** 0

        functional_groups["Ketone"] **=** smi**.**count("C(=O)") **if** "C(=O)" **in** smi **else** 0

        functional_groups["Ether"] **=** smi**.**count("O") **if** "O" **in** smi **and** "C-O-C" **in** smi **else** 0

**return** functional_groups

**else:**

**return** None

*# Function to analyze ring systems*

**def** analyze_ring_system(**smiles**)**:**

    mol **=** Chem**.**MolFromSmiles(smiles)

**if** mol**:**

        ring_info **=** {

            "Aromatic_Rings"**:** 0**,**

            "Carbocyclic_Rings"**:** 0**,**

            "Heterocyclic_Rings"**:** 0**,**

            "Double_Bonds"**:** 0**,**

            "Triple_Bonds"**:** 0**,**

            "Aliphatic_Structures"**:** 0**,**

        }

*# Count aromatic rings*

        aromatic_rings **=** 0

**for** ring **in** mol**.**GetRingInfo()**.**AtomRings()**:**

**if** all(mol**.**GetAtomWithIdx(atom_idx)**.**GetIsAromatic() **for** atom_idx **in** ring)**:**

                aromatic_rings **+=** 1

        ring_info["Aromatic_Rings"] **=** aromatic_rings

*# Count carbocyclic and heterocyclic rings*

        carbocyclic_rings **=** 0

**for** ring **in** mol**.**GetRingInfo()**.**AtomRings()**:**

**if** all(mol**.**GetAtomWithIdx(atom_idx)**.**GetSymbol() **==** "C" **for** atom_idx **in** ring)**:**

                carbocyclic_rings **+=** 1

        ring_info["Carbocyclic_Rings"] **=** carbocyclic_rings

        ring_info["Heterocyclic_Rings"] **=** len(mol**.**GetRingInfo()**.**AtomRings()) **-** carbocyclic_rings

*# Count double bonds*

        double_bonds **=** sum(1 **for** bond **in** mol**.**GetBonds() **if** bond**.**GetBondType() **==** Chem**.**rdchem**.**BondType**.**DOUBLE)

        ring_info["Double_Bonds"] **=** double_bonds

*# Count triple bonds*

        triple_bonds **=** sum(1 **for** bond **in** mol**.**GetBonds() **if** bond**.**GetBondType() **==** Chem**.**rdchem**.**BondType**.**TRIPLE)

        ring_info["Triple_Bonds"] **=** triple_bonds

*# Count aliphatic structures*

        aliphatic_structures **=** 0

**for** atom **in** mol**.**GetAtoms()**:**

**if** atom**.**GetIsAromatic() **is** False**:**

                aliphatic_structures **+=** 1

        ring_info["Aliphatic_Structures"] **=** aliphatic_structures

**return** ring_info

**else:**

**return** None

*# Function to categorize SMILES (ring system or non-ring-based)*

**def** categorize_smiles(**smiles**)**:**

    mol **=** Chem**.**MolFromSmiles(smiles)

**if** mol**:**

*# Check if the molecule has rings*

        has_rings **=** mol**.**GetRingInfo()**.**NumRings() **>** 0

*# Determine category*

**if** has_rings**:**

**return** "Ring-Based"

**else:**

**return** "Non-Ring-Based"

**else:**

**return** "Invalid_SMILES"

*# Function to read SMILES from an Excel file*

**def** read_smiles_from_excel(**file_path**)**:**

    df **=** pd**.**read_excel(file_path)

**return** df["SMILES"]**.**dropna()**.**tolist()

*# Function to process SMILES and export results*

**def** process_smiles(**file_path,** **output_file**)**:**

*# Read SMILES from the input file*

    smiles_list **=** read_smiles_from_excel(file_path)

*# Initialize a list to store results*

    results **=** []

*# Analyze each SMILES*

**for** smiles **in** smiles_list**:**

        fg_counts **=** count_functional_groups(smiles)

        ring_info **=** analyze_ring_system(smiles)

        category **=** categorize_smiles(smiles)

**if** fg_counts **and** ring_info**:**

            combined_info **=** {******fg_counts**,** ******ring_info**,** "SMILES"**:** smiles**,** "Category"**:** category}

            results**.**append(combined_info)

**else:**

            results**.**append({"SMILES"**:** smiles**,** "Invalid_SMILES"**:** 1})

*# Create a DataFrame from results*

    df_results **=** pd**.**DataFrame(results)

*# Export results to Excel*

    df_results**.**to_excel(output_file**,** **index=**False)

    print(f"Results saved to {output_file}")

*# Main script*

**if** __name__ **==** "__main__"**:**

*# Input and output file paths*

    input_file **=** r"your_pathway_to_input_file.xlsx"

    output_file **=** r"your_pathway_to_ouput_file.xlsx"

*# Process SMILES and save results*

    process_smiles(input_file**,** output_file)

1. **Random Forest model using R**

library(readr)

library(dplyr)

library(randomForest)

# Select only the functional group columns

functional_group_columns <- c("Alcohol", "Aldehyde", "Carboxylic_Acid", "Amine",

"Phenol", "Ketone", "Ether",

"Aromatic_Rings", "Carbocyclic_Rings",

"Heterocyclic_Rings", "Double_Bonds", "Triple_Bonds")

# Check which columns have more than one unique value

non_constant_columns <- apply(data[, functional_group_columns], 2,

function(x) length(unique(x)) > 1)

# Get the names of the non-constant columns (those with more than 1 unique value)

non_constant_column_names <- names(non_constant_columns)[non_constant_columns]

# Subset the data to include non-constant functional group columns and 'MIC'

data_dw_clean <- data[, c(non_constant_column_names, "MIC")]

functional_group_columns <- c("Alcohol", "Aldehyde", "Carboxylic_Acid", "Amine",

"Phenol", "Ketone",

"Aromatic_Rings", "Carbocyclic_Rings",

"Heterocyclic_Rings", "Double_Bonds")

X_train <- data_dw_clean[, functional_group_columns, drop = FALSE]

functional_group_columns <- intersect(functional_group_columns, colnames(data_dw_clean))

X_train <- data_dw_clean[, functional_group_columns, drop = FALSE]

# Define predictor

predictor <- Predictor$new(model = rf_model, data = X_train, y = data_dw_clean$MIC)

# Calculate feature importance using SHAP

shap_values <- FeatureImp$new(predictor, loss = "mse")

print(shap_values)

**Table S1.** Protein targets for molecular docking studies and the accuration of docking protocol (RMSD of native ligands)

| No | M. tuberculosis protein target | Targeted pathways | PDB ID | RMSD native ligand | Ref |
| --- | --- | --- | --- | --- | --- |
| 1 | Decaprenylphosphoryl-β-d-ribofuranose oxidoreductase (DprE1) | Cell wall biosynthesis: arabinogalactan biosynthesis | 4P8C | 0.383 | ^7^ |
| 2 | Mycolic acid cyclopropane synthase (CmaA2) | Cell wall biosynthesis: mycolic acid biosynthesis | 1KPI | 0.494 | ^8^ |
| 3 | Alanine racemase (alr) | Cell wall biosynthesis: peptidoglycan biosynthesis | 1XFC | 0.665 | ^9^ |
| 4 | 3-oxoacyl-[acyl-carrier-protein] synthase 3 (FabH) | Fatty acid biosynthesis | 1HZP | 0.998 | ^10^ |
| 5 | leucyl-tRNA synthase (LeuRS) | Protein synthesis | 5AGS | 1.023 | ^11^ |
| 6 | Protein kinase A (PknA) | Signal transduction | 6B2Q | 1.249 | ^12^ |
| 7 | Pantothenate kinase (PanK, type 1) | Cofactor biosynthesis: Coenzyme A biosynthesis | 4BFZ | 0.927 | ^13^ |
| 8 | 5’-pyridoxal phosphate (PLP)-dependent aminotransferase (BioA) | Cofactor biosynthesis: biotin biosynthesis | 4XJO | 0.050 | ^14^ |
| 9 | Aspartate aminotransferase (aspAT) | Asp biosynthesis, and Asp-dependent nitrogen metabolism | 6U7A | 1.684 | ^15^ |
| 10 | N-terminal domain of Mycobacterium tuberculosis ClpC1 | substrate recognition and unfolding | 3WDB | 0.000 | ^16^ |

**Table S2.** The result of re-ranked data after XGBoost learning.

| **Compound** | Protein Target | | | | | | | | | | | | | | | | | | | | | | |
| --- | --- | --- | --- | --- | --- | --- | --- | --- | --- | --- | --- | --- | --- | --- | --- | --- | --- | --- | --- | --- | --- | --- | --- |
|  | 1HZP | | 1KPI | | | 1XFC | | | 3WDB | | | 4BFZ | | 4P8C | | 4XJO | | 5AGS | | 6B2Q | | 6U7A | |
|  | Vina | Re-ranked | Vina | Re-ranked | Vina | | Re-ranked | Vina | | Re-ranked | Vina | | Re-ranked | Vina | Re-ranked | Vina | Re-ranked | Vina | Re-ranked | Vina | Re-ranked | Vina | Re-ranked |
| 6-hydroxymanzamine E | -9.5 | -8.9 | -8.3 | -8.7 | -8.4 | | -8.1 | -9.3 | | -9.0 | -9.0 | | -9.7 | -10.9 | -9.9 | -10.9 | -11.1 | -8.7 | -9.0 | -8.8 | -8.7 | -8.2 | -8.2 |
| 8-hydroxymanzamine A | -8.4 | -8.9 | -9.0 | -8.7 | -8.3 | | -8.2 | -9.2 | | -9.0 | -10.3 | | -9.8 | -11.4 | -10.6 | -10.7 | -11.1 | -8.9 | -9.1 | -8.2 | -8.7 | -8.3 | -8.3 |
| Manzamine E | -9.2 | -9.0 | -8.6 | -8.7 | -8.0 | | -8.2 | -9.2 | | -9.0 | -9.0 | | -9.8 | -11.3 | -10.7 | -11.4 | -11.3 | -9.6 | -9.1 | -8.7 | -8.8 | -8.2 | -8.3 |
| Manzamine A | -9.0 | -9.3 | -9.1 | -9.0 | -8.4 | | -8.4 | -9.3 | | -9.3 | -9.9 | | -10.0 | -11.9 | -11.4 | -11.4 | -11.7 | -9.0 | -9.4 | -8.3 | -9.7 | -8.4 | -8.5 |
| Manzamine F | -9.1 | -9.1 | -8.7 | -8.8 | -8.2 | | -8.2 | -8.5 | | -9.1 | -9.1 | | -9.8 | -12.2 | -10.9 | -11.0 | -11.3 | -9.7 | -9.1 | -8.6 | -8.9 | -8.2 | -8.3 |
| Manzamine A-*N*-oxide | -8.5 | -8.8 | -8.6 | -8.6 | -8.2 | | -8.0 | -8.3 | | -8.9 | -9.8 | | -9.6 | -11.6 | -9.7 | -11.1 | -11.0 | -9.0 | -9.0 | -8.1 | -8.6 | -8.3 | -8.2 |
| *ent*-8-hydroxymanzamine A | -8.6 | -9.4 | -8.4 | -9.1 | -8.9 | | -8.8 | -10.2 | | -9.6 | -9.0 | | -10.4 | -11.8 | -11.8 | -11.8 | -12.6 | -9.6 | -9.5 | -8.7 | -9.9 | -8.3 | -8.6 |
| Neo-kauluamine | -12.1 | -11.5 | -10.6 | -10.6 | -10.7 | | -10.8 | -10.2 | | -10.3 | -12.0 | | -11.7 | -9.8 | -11.8 | -12.6 | -12.6 | -9.0 | -9.5 | -9.1 | -10.2 | -10.7 | -10.7 |
| Manzamine J | -9.2 | -9.2 | -9.2 | -9.0 | -8.0 | | -8.3 | -9.0 | | -9.1 | -9.8 | | -9.9 | -11.9 | -11.4 | -11.2 | -11.7 | -9.2 | -9.3 | -8.5 | -9.5 | -8.5 | -8.4 |
| 6-deoxy-manzamine X | -9.4 | -9.3 | -9.0 | -9.0 | -8.8 | | -8.8 | -9.4 | | -9.4 | -10.0 | | -10.1 | -9.3 | -11.6 | -11.9 | -12.3 | -9.6 | -9.4 | -9.0 | -9.7 | -8.5 | -8.5 |
| Ircinol | -7.6 | -7.5 | -7.2 | -6.7 | -6.3 | | -6.3 | -7.8 | | -7.5 | -6.9 | | -8.3 | -8.7 | -8.0 | -8.6 | -8.5 | -6.9 | -7.2 | -6.2 | -7.1 | -6.5 | -6.5 |
| Batzelladine L | -8.2 | -7.7 | -7.5 | -7.1 | -7.0 | | -6.5 | -6.9 | | -7.5 | -8.6 | | -8.4 | -7.8 | -8.0 | -8.7 | -8.7 | -7.5 | -7.3 | -7.8 | -7.4 | -5.8 | -6.8 |
| Batzelladine N | -7.9 | -7.9 | -7.4 | -7.4 | -7.6 | | -6.8 | -7.9 | | -7.7 | -8.1 | | -8.5 | -9.3 | -8.5 | -8.7 | -8.8 | -7.8 | -7.5 | -7.4 | -7.4 | -7.0 | -6.9 |
| Denigrin C | -9.4 | -9.1 | -8.9 | -8.8 | -7.6 | | -8.3 | -9.1 | | -9.1 | -10.6 | | -9.8 | -10.8 | -11.4 | -13.1 | -11.3 | -8.7 | -9.2 | -8.4 | -9.3 | -7.2 | -8.4 |
| Ascididemin | -8.8 | -8.5 | -7.5 | -8.2 | -6.6 | | -7.7 | -8.0 | | -8.4 | -9.3 | | -9.2 | -9.1 | -9.2 | -10.1 | -9.8 | -8.9 | -8.3 | -10.0 | -8.1 | -7.2 | -7.6 |
| Kuanoniamine A | -8.5 | -8.2 | -7.2 | -7.5 | -6.3 | | -7.0 | -7.8 | | -8.0 | -8.8 | | -8.7 | -8.9 | -8.8 | -9.0 | -9.3 | -8.3 | -7.7 | -9.5 | -7.6 | -6.9 | -7.2 |
| Heteronemin | -8.5 | -8.5 | -8.7 | -8.1 | -7.6 | | -7.7 | -8.4 | | -8.4 | -9.6 | | -9.1 | -9.7 | -9.1 | -10.2 | -9.8 | -7.4 | -8.3 | -7.4 | -8.1 | -7.7 | -7.5 |
| 12-deacetoxyscalarin-19-acetate | -8.8 | -8.7 | -8.7 | -8.5 | -8.0 | | -7.9 | -9.0 | | -8.8 | -9.1 | | -9.4 | -9.6 | -9.6 | -9.8 | -10.8 | -8.1 | -8.9 | -8.1 | -8.6 | -7.3 | -8.1 |
| Heteronemin acetate | -8.0 | -8.0 | -7.1 | -7.5 | -7.5 | | -6.9 | -7.7 | | -7.8 | -9.7 | | -8.7 | -8.7 | -8.8 | -9.6 | -9.3 | -7.4 | -7.6 | -7.6 | -7.6 | -6.9 | -7.2 |
| 12-oxoheteronemin | -8.6 | -8.7 | -9.1 | -8.5 | -7.9 | | -7.8 | -8.7 | | -8.6 | -9.9 | | -9.3 | -9.6 | -9.6 | -9.9 | -10.3 | -7.2 | -8.9 | -7.4 | -8.5 | -8.0 | -8.1 |
| Decarine | -8.3 | -8.4 | -7.8 | -7.9 | -6.8 | | -7.4 | -8.3 | | -8.2 | -9.1 | | -8.9 | -9.1 | -9.0 | -9.4 | -9.5 | -7.9 | -8.0 | -10.3 | -7.8 | -7.2 | -7.3 |
| 2'-nortiliacorinine | -8.0 | -8.8 | -8.4 | -8.6 | -8.1 | | -8.0 | -9.1 | | -8.9 | -8.6 | | -9.6 | -10.7 | -9.7 | -10.7 | -10.9 | -8.7 | -9.0 | -11.1 | -8.6 | -8.0 | -8.1 |
| Tiliacorine | -7.9 | -8.7 | -8.1 | -8.5 | -7.7 | | -7.8 | -8.0 | | -8.6 | -8.6 | | -9.4 | -9.7 | -9.6 | -11.4 | -10.6 | -8.6 | -8.9 | -7.9 | -8.5 | -8.4 | -8.1 |
| 13'-bromo-tiliacorinine | -7.5 | -8.7 | -8.5 | -8.4 | -7.7 | | -7.7 | -8.8 | | -8.6 | -8.6 | | -9.3 | -8.2 | -9.3 | -10.6 | -10.0 | -8.1 | -8.6 | -9.9 | -8.2 | -8.1 | -7.8 |
| Globospiramine | -8.4 | -8.6 | -9.1 | -8.2 | -8.2 | | -7.7 | -8.6 | | -8.5 | -8.5 | | -9.3 | -8.9 | -9.2 | -11.1 | -9.8 | -7.7 | -8.5 | -7.3 | -8.2 | -8.2 | -7.6 |
| Isogoyazensolide | -7.8 | -7.6 | -6.6 | -6.8 | -5.9 | | -6.4 | -7.6 | | -7.5 | -8.5 | | -8.4 | -7.8 | -8.0 | -8.5 | -8.6 | -6.8 | -7.2 | -7.2 | -7.2 | -8.2 | -6.6 |
| Isocentratherin | -6.9 | -7.3 | -6.3 | -6.5 | -6.4 | | -6.2 | -7.0 | | -7.1 | -8.3 | | -8.3 | -7.6 | -7.9 | -8.1 | -8.3 | -7.6 | -7.0 | -7.9 | -7.0 | -6.2 | -6.2 |
| 5-*epi*-isogoyazensolide | -8.2 | -7.2 | -7.1 | -6.3 | -6.2 | | -6.1 | -6.7 | | -6.9 | -7.9 | | -7.4 | -7.9 | -7.7 | -7.8 | -7.9 | -6.8 | -6.9 | -6.1 | -6.4 | -5.9 | -6.1 |
| 5-*epi*-isocentratherin | -7.1 | -7.5 | -6.4 | -6.7 | -6.9 | | -6.3 | -7.1 | | -7.2 | -8.4 | | -8.3 | -8.5 | -7.9 | -8.3 | -8.4 | -7.7 | -7.0 | -6.4 | -7.0 | -5.9 | -6.4 |
| Goyazensolide | -6.9 | -7.2 | -6.1 | -6.4 | -5.9 | | -6.1 | -7.9 | | -7.0 | -8.5 | | -8.0 | -7.7 | -7.8 | -8.3 | -8.2 | -6.9 | -7.0 | -6.5 | -6.5 | -6.4 | -6.1 |
| Lychnophorolide A | -7.1 | -7.2 | -6.1 | -6.5 | -5.8 | | -6.2 | -7.8 | | -7.1 | -8.3 | | -8.0 | -8.0 | -7.9 | -8.6 | -8.2 | -7.0 | -7.0 | -7.1 | -6.9 | -6.1 | -6.1 |
| Plumbagin | -7.5 | -7.2 | -8.0 | -6.3 | -5.8 | | -6.1 | -6.4 | | -6.8 | -6.5 | | -7.2 | -7.8 | -7.6 | -6.6 | -7.5 | -6.9 | -6.7 | -7.2 | -6.4 | -7.1 | -6.1 |
| Maritinone | -9.0 | -8.4 | -7.9 | -7.9 | -6.8 | | -7.2 | -8.5 | | -8.2 | -8.9 | | -8.8 | -9.0 | -9.0 | -9.2 | -9.4 | -9.4 | -7.9 | -7.1 | -7.7 | -6.6 | -7.3 |
| 3,3'-biplumbagin | -8.7 | -8.5 | -7.8 | -7.9 | -7.1 | | -7.5 | -8.3 | | -8.3 | -9.8 | | -9.0 | -9.4 | -9.1 | -9.4 | -9.6 | -9.5 | -8.2 | -7.3 | -8.0 | -7.3 | -7.4 |
| Lobophorin B | -6.6 | -8.0 | -6.3 | -7.4 | -7.4 | | -6.8 | -8.0 | | -7.7 | -9.6 | | -8.6 | -8.0 | -8.5 | -9.8 | -9.0 | -7.1 | -7.5 | -7.9 | -7.5 | -9.1 | -6.9 |
| Lobophorin C | -8.7 | -7.9 | -6.3 | -7.4 | -7.1 | | -6.7 | -6.5 | | -7.6 | -9.4 | | -8.5 | -8.7 | -8.3 | -8.5 | -8.7 | -7.8 | -7.4 | -6.8 | -7.4 | -7.5 | -6.9 |
| Lobophorin I | -8.3 | -8.3 | -8.3 | -7.9 | -8.5 | | -7.1 | -8.2 | | -8.0 | -7.5 | | -8.7 | -7.5 | -8.9 | -9.9 | -9.4 | -6.0 | -7.7 | -8.8 | -7.6 | -8.3 | -7.2 |
| Urdamycinone E | -9.4 | -9.1 | -9.1 | -8.9 | -7.9 | | -8.3 | -8.5 | | -9.1 | -9.5 | | -9.8 | -9.6 | -11.4 | -12.5 | -11.3 | -8.7 | -9.2 | -10.5 | -9.4 | -8.5 | -8.4 |
| Gliotoxin | -7.0 | -7.0 | -6.2 | -6.2 | -5.2 | | -6.0 | -7.2 | | -6.8 | -7.5 | | -7.0 | -6.6 | -6.9 | -7.0 | -7.1 | -6.7 | -6.5 | -6.4 | -6.4 | -5.7 | -5.8 |
| Fumitremorgin C | -9.0 | -8.0 | -7.4 | -7.4 | -6.8 | | -6.9 | -7.5 | | -7.8 | -8.6 | | -8.6 | -8.9 | -8.7 | -9.3 | -9.2 | -7.9 | -7.5 | -8.1 | -7.6 | -6.4 | -7.0 |
| Deoxypreussomerin A | -8.9 | -8.5 | -8.8 | -8.0 | -6.9 | | -7.5 | -8.6 | | -8.4 | -8.9 | | -9.1 | -8.9 | -9.1 | -9.3 | -9.7 | -9.2 | -8.3 | -7.6 | -8.1 | -7.6 | -7.5 |
| Preussomerin F | -8.6 | -8.7 | -7.9 | -8.4 | -7.7 | | -7.8 | -9.1 | | -8.6 | -10.4 | | -9.3 | -9.0 | -9.3 | -9.1 | -10.0 | -9.4 | -8.8 | -7.9 | -8.4 | -7.0 | -7.8 |
| Preussomerin G | -8.8 | -8.8 | -8.1 | -8.6 | -7.7 | | -8.0 | -9.2 | | -8.8 | -10.2 | | -9.4 | -9.2 | -9.7 | -9.6 | -10.8 | -9.7 | -8.9 | -8.8 | -8.6 | -7.2 | -8.1 |
| Trichoderin A | -7.1 | -7.0 | -5.6 | -6.0 | -6.1 | | -5.8 | -5.8 | | -5.1 | -7.0 | | -6.7 | -7.0 | -6.6 | -6.5 | -6.7 | -6.1 | -6.3 | -6.4 | -6.2 | -5.3 | -5.5 |
| Trichoderin A1 | -7.1 | -7.0 | -6.1 | -6.2 | -6.2 | | -6.0 | -5.0 | | -5.8 | -6.6 | | -6.9 | -6.5 | -6.7 | -7.6 | -6.7 | -6.4 | -6.4 | -6.2 | -6.3 | -5.4 | -5.8 |
| Trichoderin B | -6.5 | -6.6 | -6.5 | -5.7 | -6.4 | | -5.3 | -4.2 | | -4.7 | -7.4 | | -6.7 | -6.3 | -6.4 | -6.7 | -6.6 | -6.3 | -6.1 | -6.1 | -6.1 | -6.0 | -5.5 |

**References**

1 T. Sander, J. Freyss, M. Von Korff and C. Rufener, *J Chem Inf Model*, 2015, **55**, 460–473.

2 V. Svetnik, A. Liaw, C. Tong, J. Christopher Culberson, R. P. Sheridan and B. P. Feuston, *J Chem Inf Comput Sci*, 2003, **43**, 1947–1958.

3 T. X. Trinh, M. Seo, T. H. Yoon and J. Kim, *NanoImpact*, 2022, **25**, 100383.

4 O. Trott and A. J. Olson, *J Comput Chem*, 2009, **31**, 455–461.

5 A. Y. Bande and S. Baday, *Mol Inform*, 2024, **43**, e202300167.

6 C. Yang and Y. Zhang, *J Chem Inf Model*, 2022, **62**, 2696–2712.

7 M. N. Al-Qattan, P. K. Deb and R. K. Tekade, *Drug Discov Today*, 2018, **23**, 235–250.

8 P. K. Deb, N. A. Al-Shar’i, K. N. Venugopala, M. Pillay and P. Borah, *J Enzyme Inhib Med Chem*, 2021, **36**, 869–884.

9 P. LeMagueres, H. Im, J. Ebalunode, U. Strych, M. J. Benedik, J. M. Briggs, H. Kohn and K. L. Krause, *Biochemistry*, 2005, **44**, 1471–1481.

10 D. Portevin, C. Lia De Sousa-D’auria, C. Houssin, C. Grimaldi, M. Chami, M. Daffé, C. Guilhot and ¶ † Dé Partement ’ ’mé, *PNAS*, 2004, **101**, 314–319.

11 J. Li, A. Fu and L. Zhang, *Interdiscip Sci*, 2019, **11**, 320–328.

12 C. M. Kang, D. W. Abbott, T. P. Sang, C. C. Dascher, L. C. Cantley and R. N. Husson, *Genes Dev*, 2005, **19**, 1692–1704.

13 L. R. Chiarelli, G. Mori, B. S. Orena, M. Esposito, T. Lane, A. L. De Jesus Lopes Ribeiro, G. Degiacomi, J. Zemanová, S. Szádocka, S. Huszár, Z. Palčeková, M. Manfredi, F. Gosetti, J. Lelièvre, L. Ballell, E. Kazakova, V. Makarov, E. Marengo, K. Mikusova, S. T. Cole, G. Riccardi, S. Ekins and M. R. Pasca, *Sci Rep*, 2018, **8**, 3187.

14 F. Liu, S. Dawadi, K. M. Maize, R. Dai, S. W. Park, D. Schnappinger, B. C. Finzel and C. C. Aldrich, *J Med Chem*, 2017, **60**, 5507–5520.

15 R. S. Jansen, L. Mandyoli, R. Hughes, S. Wakabayashi, J. T. Pinkham, B. Selbach, K. M. Guinn, E. J. Rubin, J. C. Sacchettini and K. Y. Rhee, *Nat Commun*, 2020, **11**, 1960.

16 N. M. Wolf, H. Lee, D. Zagal, J. W. Nam, D. C. Oh, H. Lee, J. W. Suh, G. F. Pauli, S. Cho and C. Abad-Zapatero, *Acta Crystallogr D Struct Biol*, 2020, **76**, 458–471.
